# Ion-Beam-Induced Atomic Mixing in Ge, Si, and SiGe, Studied by Means of Isotope Multilayer Structures

**DOI:** 10.3390/ma10070813

**Published:** 2017-07-17

**Authors:** Manuel Radek, Bartosz Liedke, Bernd Schmidt, Matthias Voelskow, Lothar Bischoff, John Lundsgaard Hansen, Arne Nylandsted Larsen, Dominique Bougeard, Roman Böttger, Slawomir Prucnal, Matthias Posselt, Hartmut Bracht

**Affiliations:** 1 Institute of Materials Physics, Westfälische Wilhelms-Universität Münster, 48149 Münster, Germany; manuel.radek@uni-muenster.de; 2 Helmholtz-Zentrum Dresden-Rossendorf, 01328 Dresden, Germany; liedkeb@gmail.com (B.L.); bernd.schmidt@hzdr.de (B.S.); m.voelskow@hzdr.de (M.V.); l.bischoff@hzdr.de (L.B.); r.boettger@hzdr.de (R.B.); s.prucnal@hzdr.de (S.P.); 3 Department of Physics and Astronomy, Aarhus University, 8000 Aarhus, Denmark; johnlh@phys.au.dk (J.L.H.); anl@phys.au.dk (A.N.L.); 4 Institut für Experimentelle und Angewandte Physik, Universität Regensburg, 93040 Regensburg, Germany; dominique.bougeard@physik.uni-regensburg.de

**Keywords:** silicon, germanium, ion beam, atomic mixing, thermal spike, radiation enhanced diffusion, amorphization, recrystallization, molecular dynamics

## Abstract

Crystalline and preamorphized isotope multilayers are utilized to investigate the dependence of ion beam mixing in silicon (Si), germanium (Ge), and silicon germanium (SiGe) on the atomic structure of the sample, temperature, ion flux, and electrical doping by the implanted ions. The magnitude of mixing is determined by secondary ion mass spectrometry. Rutherford backscattering spectrometry in channeling geometry, Raman spectroscopy, and transmission electron microscopy provide information about the structural state after ion irradiation. Different temperature regimes with characteristic mixing properties are identified. A disparity in atomic mixing of Si and Ge becomes evident while SiGe shows an intermediate behavior. Overall, atomic mixing increases with temperature, and it is stronger in the amorphous than in the crystalline state. Ion-beam-induced mixing in Ge shows no dependence on doping by the implanted ions. In contrast, a doping effect is found in Si at higher temperature. Molecular dynamics simulations clearly show that ion beam mixing in Ge is mainly determined by the thermal spike mechanism. In the case of Si thermal spike, mixing prevails at low temperature whereas ion beam-induced enhanced self-diffusion dominates the atomic mixing at high temperature. The latter process is attributed to highly mobile Si di-interstitials formed under irradiation and during damage annealing.

## 1. Introduction

Ion implantation and subsequent thermal processing are frequently used methods for doping of semiconductors. Ion irradiation induces a redistribution of atoms, which is generally referred to as ion-beam-induced atomic mixing or ion beam mixing. With the decreasing size of functional materials, the precise prediction and controlling of dopant distribution at the nanoscale becomes increasingly important. Furthermore, state-of-the-art sputtering and ion beam techniques used for characterization of dopant profiles and interfaces between different materials also induce atomic mixing, which limits the achievable depth resolution. Therefore, the understanding of ion beam mixing is not only crucial for the optimization of semiconductor processing, but also for the evaluation of the depth resolution of state-of-the-art sputtering techniques like, e.g., secondary ion mass spectrometry.

Over the past decades ion beam mixing was mainly investigated indirectly as a result of irradiation-induced broadening of impurity profiles in metals and silicon at low temperatures [1]. These investigations indicate, that ion beam mixing is a very complex process [1,2]. It depends not only on the kinetic energy and mass of the impinging ions, but also on the thermal conductivity of the target material and the sample temperature during implantation. Furthermore, chemical processes strongly affect the mixing of heterogeneous systems, i.e., systems with a negative heat of mixing reveal stronger mixing than systems with a positive heat of mixing [3,4]. The present work is focused on the investigation of ion-beam-induced atomic mixing in elemental semiconductor materials Si and Ge where chemical effects do not play any role. Additionally, the compound semiconductor SiGe is investigated. The complete miscibility of this material suggests that chemical effects with regard to the intermixing of matrix atoms are of minor importance. The insignificance of chemical effects allows for a good comparability to theoretical and numerical investigations on physical effects of atomic mixing. However, investigations of atomic mixing in a homogeneous material can only be achieved by labelling the matrix atoms. With the development of epitaxial deposition techniques and the availability of stable isotopes, manufacturing of isotopically controlled Si and Ge multilayer structures became possible. These structures enabled numerous fundamental studies that significantly profit from an isotopic labelling of matrix atoms, such as the interference of self- and dopant diffusion [5,6].

Irradiation with ions in the keV range creates energetic primary knock-on atoms within the target material, provided the energy transferred during the collision exceeds the material dependent displacement energy (typically 10–40 eV). These primary recoils can create second-order recoils with lower energy, which may produce even higher order recoils, leading to a collision cascade. With decreasing energy the collisions become more localized resulting in regions with high energy deposition and atomic disorder. This can cause a “thermal spike”, a notation that is often used to describe collective interactions between atoms in the region of a dense cascade [1]. The thermal spike region is characterized by a temperature determined by the deposited kinetic energy. This temperature can even exceed the melting point of the target material and causes atomic transport that is significantly faster than in the solid phase. In the 1950s Brinkman et al. [7] were the first to discuss the concept of thermal spikes induced by impinging ions. First theoretical investigations of atomic mixing induced by thermal spikes were performed almost 30 years later in the early 1980s using molecular dynamics simulation. Webb and Harrison [8] showed that thermal spike effects can clearly enhance atomic mixing. Due to the limited computational resources only a small number of atoms could be simulated and the results were only interpreted qualitatively. In 1987 Diaz de la Rubia et al. [9] used larger simulation cells and obtained a reasonably good agreement with experimental diffusion coefficients in the melt, proving that the basic idea of Brinkman et al. [7] is valid. In the late 1990s Nordlund et al. [10] performed a comprehensive molecular dynamics study on atomic mixing in several metals and semiconductors, revealing significant differences in the atomic mixing of these materials. However, all these simulations and most of the experiments on ion beam mixing were performed at low temperatures to suppress additional effects such as radiation enhanced diffusion that complicate the analysis of atomic mixing. Furthermore, only limited comparability to experimental results is achieved by the simulations due to the lack of appropriate sample structures. The combination of isotopically modulated structures and state-of-the-art molecular dynamics simulation is therefore a promising toolset to understand the atomic transport mechanisms during ion irradiation in Si and Ge at various temperatures.

In the last three years various results on investigations of ion beam mixing in Ge [11] and Si [12] have been already published. The present work contains a comprehensive summary of our research project. Within its framework we directly measured ion-beam-induced atomic mixing at various temperatures in Si, Ge and SiGe by means of isotopically enriched structures. A comparison between atomic mixing in crystalline and preamorphized structures gives a measure on the structural dependence of atomic mixing. Additionally, varying doping conditions are realized in the experiments. The experimentally obtained atomic mixing is compared to state-of-the-art molecular dynamics simulations. Because of the complex nature of ion beam mixing a large amount of individual simulations and a statistical analysis is necessary for a consistent comparison with experimental results. The present work is organized in the following manner: In the first part the experiments on ion beam mixing in Ge, Si and SiGe are described, and the dependence of the results on implantation temperature, ion flux, and ion species are discussed. In the second part molecular dynamics simulations and reaction-diffusion equations are employed to interpret the experimental data more quantitatively and on the atomistic level. Finally, the processes of ion-beam-induced atomic mixing in Ge and Si are compared and the results of the work are summarized.

## 2. Ion-Beam-Induced Atomic Mixing in Germanium

### 2.1. Temperature Dependence—${}^{69}$Ga and ${}^{70}$Ge Implantation

#### 2.1.1. Experimental

**Figure 1:**
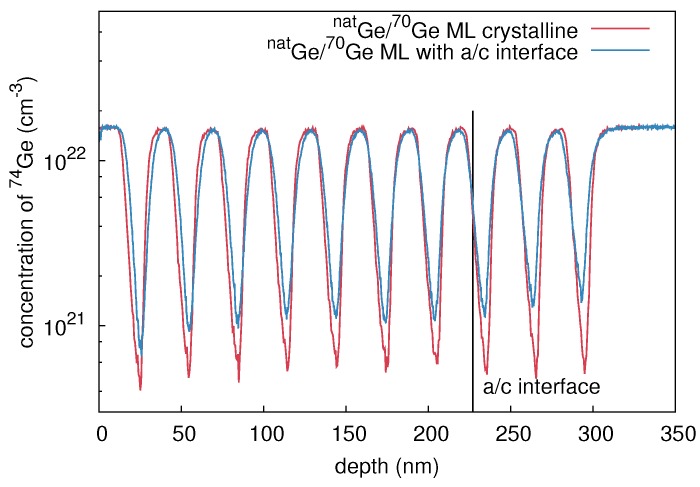
${}^{74}$Ge concentration depth profiles measured with secondary ion mass spectrometry (SIMS) before (red) and after preamorphization (blue) by 310 keV ${}^{70}$Ge ions at an ion fluence of $7\times 10^{13}$ cm${}^{-2}$. The molecular beam epitaxy (MBE) grown multilayer stack consists of 10 bilayers of ${}^{\mathrm{nat}}$Ge and ${}^{70}$Ge with an individual layer thickness of about 15 nm.

A stack of ten (mono)crystalline bilayers (${}^{\mathrm{nat}}$Ge/${}^{70}$Ge)${}_{10}$ was grown by means of molecular beam epitaxy (MBE). The thickness of each natural and isotopically enriched layer is about 15 nm. The crystalline structure was deposited on a (100)-oriented n-type Ge wafer with a specific resistivity of 40 Ωcm. Amorphous multilayer structures were prepared by ${}^{70}$Ge ion implantation into the crystalline isotope structure. The implantation was performed at 77 K with 310 keV ${}^{70}$Ge${}^{+}$ ions, a fluence of 7 × 10${}^{13}$ cm${}^{-2}$, and an ion current of 200 nA. The amorphization fluence of ${}^{70}$Ge at 77 K is about 6 × 10${}^{13}$ cm${}^{-2}$, according to Koffel et al. [13]. Cross-sectional transmission electron microscope (XTEM) measurements of these samples reveal complete amorphization up to a depth of about 230 nm. Amorphization at low temperatures ensures that no voids or cavities are built during the implantation process, which are known to form at temperatures between 220 K and 470 K or high ion fluences in Ge [14]. Even under the conditions of our ion-beam mixing experiments a void formation is not observed. This is in accordance to the reports on void formation observed for higher fluences than applied in our work [13,15,16,17]. However, a 50 nm wide defective range is observed beneath the amorphous Ge layer. Figure 1 illustrates ${}^{74}$Ge concentration profiles of the as-grown crystalline (c-Ge: red lines) and preamorphized (a-Ge: blue lines) isotope structures measured with secondary ion mass spectrometry (SIMS). The SIMS measurements were performed with a time-of-flight-SIMS-5 system in a dual beam mode. Oxygen ions with 1 keV energy were used for sputtering and 25 keV bismuth ions for analysis. The depth of the craters left from the SIMS analysis was determined with an optical profilometer. The sputter time was converted to penetration depths taking into account the crater depth and assuming a constant sputter rate during SIMS profiling. With the well-known abundance of the Ge isotopes in natural Ge, the detected Ge ion counts were transformed to concentrations. The obtained concentration profiles of ${}^{74}$Ge are analytically described by

(1) $$C_{Ge}\left(x\right)=C_{1}+\frac{C_{2}-C_{1}}{2}\sum_{i=1}^{20}{\left(-1\right)}^{i+1}\mathrm{erfc}\left(\frac{x-x_{i}}{r_{i}}\right),$$

where *x* is the depth, $C_{1}$ and $C_{2}$ the minimum and maximum concentrations of the ${}^{74}$Ge isotope, and erfc(*x*) = 1-erf(x) is the complementary error function. The error function erf(*x*) has the asymptotic behavior erf(−∞) = -1 and erf(∞) = +1. $x_{i}$ represents the depth of the *i*th isotope interface and $r_{i}$ is a measure of the steepness of the Ge concentration at the *i*th interface between natural and isotopically enriched Ge layers.

In order to study ion-beam-induced atomic mixing, 310 keV ${}^{69}$Ga${}^{+}$ ions were implanted with a fluence of 1 × 10${}^{15}$ cm${}^{-2}$ and an ion current of 200 nA at six different temperatures (164, 219, 300, 423, 523, 623 K), whereas ${}^{70}$Ge${}^{+}$ ions were implanted at 723 and 823 K using same energy, fluence and current. After implantation, the temperature was rapidly reduced by liquid nitrogen cooling to suppress any post-implantation induced self-atom mixing that could be caused by the dissolution of defect clusters formed during implantation. Due to the construction of the target chamber, all implantations were performed at nearly normal incidence to the (100) sample surface. At 164 and 219 K (723 and 823 K), ${}^{69}$Ga (Ge) implantation was only performed into initially crystalline Ge isotope structures, whereas both crystalline and preamorphized samples were implanted simultaneously at the other temperatures. The implantation damage was investigated by Rutherford backscattering spectrometry in channeling geometry (RBS/C). RBS/C was performed using a collimated 1.7 MeV He${}^{+}$ beam and the sample was mounted on a five-axis, high precision (±0.01${}^{\circ }$) goniometer.

#### 2.1.2. Results and Discussion

**Figure 2:**
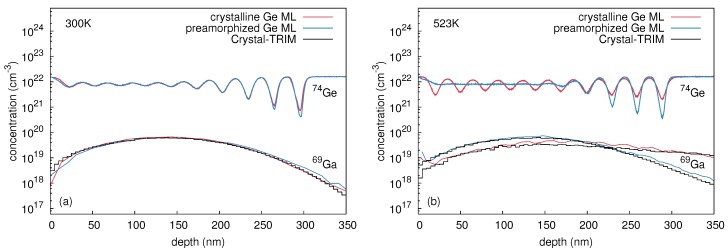
Concentration profiles of ${}^{74}$Ge and ${}^{69}$Ga in initially crystalline (red) and preamorphized (blue) (${}^{\mathrm{nat}}$Ge/${}^{70}$Ge)${}_{10}$ multilayer structures measured by SIMS after implantation with 310 keV ${}^{69}$Ga at a fluence of 1 × 10${}^{15}$ cm${}^{-2}$ and at temperatures of (**a**) 300 K and (**b**) 523 K. Additionally, Ga implantation profiles as predicted by Crystal-TRIM are shown (black histogramms). Reproduced from [Radek, M.; Bracht, H.; Posselt, M.; Liedke, B.; Schmidt, B.; Bougeard, D. Temperature dependence of ion-beam mixing in crystalline and amorphous germanium isotope multilayer structures. *J. Appl. Phys.* **2014**, *115*, 023506.], with the permission of AIP Publishing.

Figure 2 shows concentration profiles of ${}^{74}$Ge and ${}^{69}$Ga measured with SIMS, after ${}^{69}$Ga ion implantation into initially crystalline (c-Ge: red lines) and preamorphized (a-Ge: blue lines) Ge isotope structures at 300 and 523 K. At 300 K self-atom mixing in c-Ge, indicated by the ${}^{74}$Ge concentration profiles in Figure 2a, is very similar to the mixing in a-Ge. In contrast, the self-atom profiles obtained after ${}^{69}$Ga implantation at 523 K differ strongly across the whole isotope structure as illustrated by Figure 2b. A comparison between 300 and 523 K reveals that for x≤ 200 nm, the self-atom mixing in c-Ge at 300 K is stronger than at 523 K. For *x* > 200 nm, the reverse is true, i.e., the self-atom mixing in c-Ge at 300 K is weaker than at 523 K. On the other hand, the amorphous Ge structure is clearly stronger mixed at 523 K than at 300 K for x≤ 200 nm, whereas for *x* > 200 nm, the self-atom mixing of a-Ge at 300 K and 523 K is very similar.

Figure 2a demonstrates that at 300 K the Ga profile in c-Ge is very similar to the Ga profile in a-Ge. Both concentration profiles are reproduced well by Crystal-TRIM calculations (cf., e.g., Reference [18], black histograms) assuming an amorphous target, the thickness of which is larger than the amorphous layer of the preamorphized sample (400 nm). This is in accord with results of RBS/C measurements. At 523 K (see Figure 2b), the Ga profile in c-Ge strongly deviates from the Ga profile in a-Ge, but is accurately reproduced by Crystal-TRIM calculations assuming a crystalline structure during the whole ${}^{69}$Ga implantation process. The Ga profile in a-Ge follows the Crystal-TRIM prediction when a 200 nm thick amorphous layer on top of a crystalline structure is assumed. The assumptions made in Crystal-TRIM simulations are consistent with data obtained by RBS/C analysis.

The difference in self-atom mixing of c-Ge and a-Ge can be evaluated more quantitatively by modeling the implantation-induced atomic displacements with a convolution integral

(2) $$C_{\mathrm{Ge}}^{\mathrm{after}}\left(x\right)=\int C_{\mathrm{Ge}}^{\mathrm{before}}\left(x^{\prime }\right)\cdot g\left(x-x^{\prime }\right)\cdot dx^{\prime },$$

where $C_{\mathrm{Ge}}^{\mathrm{before}}\left(x\right)$ and $C_{\mathrm{Ge}}^{\mathrm{after}}\left(x\right)$ represent the Ge distribution before and after ${}^{69}$Ga implantation, respectively. *g* denotes a Gaussian function of the form

(3) $$g\left(x\right)=\frac{1}{\sqrt{2\pi \sigma }}\exp\left(-\frac{x^{2}}{2\sigma^{2}}\right).$$

The quantity σ determines the width of the Gaussian function and is assumed to be depth dependent

(4) $$\sigma \left(x\right)=k\cdot \exp\left(-\frac{{\left(x-l\right)}^{2}}{2m^{2}}\right).$$

**Figure 3:**
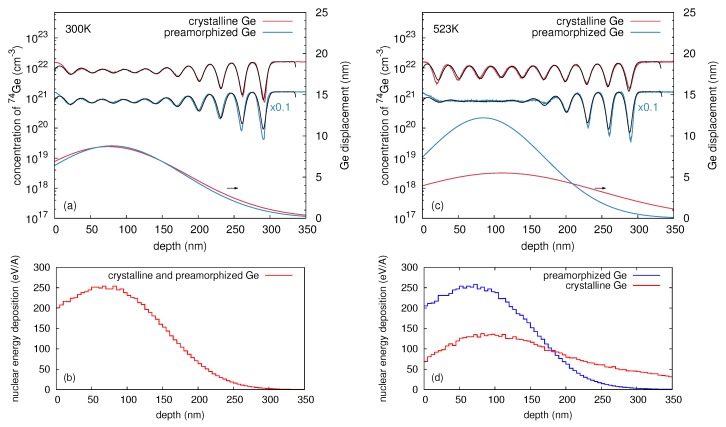
Best fits (black lines) based on Equations (2)–(4) to the implantation induced atomic mixing profiles of initially crystalline (red) and preamorphized (blue) Ge at (**a**) 300 K and (**c**) 523 K. The ${}^{74}$Ge concentration profiles of crystalline and preamorphized Ge are differently scaled for clarity. The lower solid lines show the corresponding Ge displacement function σ(x) and are referred to the right axis. The parameters of σ(x) for all samples investigated in this work are summarized in Table 1. The depth profiles of the nuclear energy deposition per target atom calculated by Crystal-TRIM are also depicted (see the corresponding (**b**,**d**) at the bottom). (**a**,**c**) were reproduced from [Radek, M.; Bracht, H.; Posselt, M.; Liedke, B.; Schmidt, B.; Bougeard, D. Temperature dependence of ion-beam mixing in crystalline and amorphous germanium isotope multilayer structures. *J. Appl. Phys.* **2014**, *115*, 023506.], with the permission of AIP Publishing.

With Equations (2)–(4) the depth dependent ion-beam induced self-atom mixing of the Ge multilayer is described by means of a depth dependent displacement function σ(x). The parameters *k*, *l* and *m* are the amplitude, position and width of the σ(x) function. These parameters were optimized to accurately describe the ${}^{74}$Ge concentration profiles and are given in Table 1. Figure 3a,c show best fits to the experimental ${}^{74}$Ge profiles obtained after ${}^{69}$Ga implantation into c- and a-Ge at 300 and 523 K. The corresponding σ(x) profiles referred to the right ordinate are also displayed. The similarity of the σ(x) profiles at 300 K (see Figure 3a) expresses the almost identical self-atom mixing in c- and a-Ge at this temperature. On the other hand, the disparity of the σ(x) profiles at 523 K (see Figure 3c) indicates the difference in self-atom mixing in c- and a-Ge. As expected, the Ge displacement function σ(x) for c- and a-Ge is in qualitative agreement with the depth profile of the nuclear energy deposition (per target atom) calculated by Crystal-TRIM (see Figure 3b,d). This quantity is a measure of the intensity of atomic collisions and, therefore, of the self-atom mixing.

**Figure 4:**
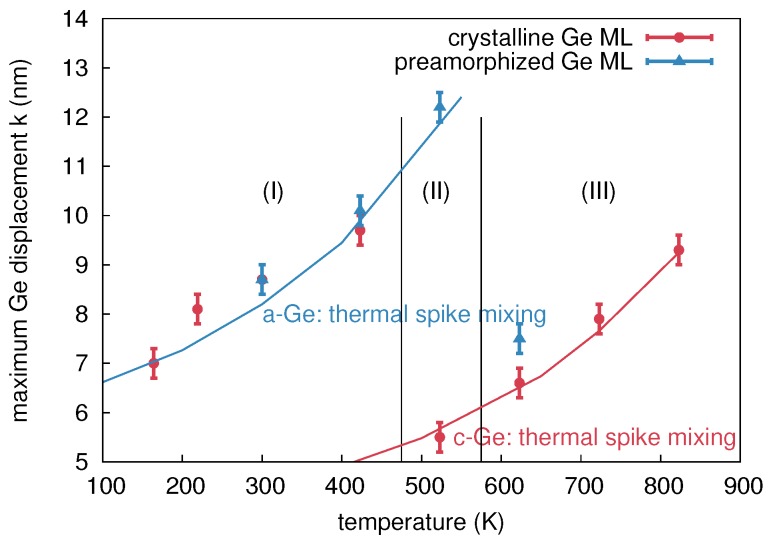
Temperature dependence of the maximum Ge displacement *k*, cf. Table 1. The data obtained for the initially crystalline (preamorphized) samples are marked in red (blue). Three temperature regimes (I, II, III) with different mixing characteristics are identified. The lines show the results of molecular dynamics simulations (cf. Section 5) of thermal spike mixing in crystalline (red) and amorphous (blue) Ge.

The amplitude of mixing *k*, i.e., the maximum Ge displacement, is shown in Figure 4 as a function of the sample temperature during ion implantation. The temperature dependence of ion beam mixing in c- and a-Ge is divided into three regions. In region I, ion beam mixing in the initially crystalline and the preamorphized Ge is nearly equal. Within this region, the Ge displacement increases with temperature. The values for c- and a-Ge at 300 and 423 K are equal within the experimental accuracy indicating that the corresponding Ge profiles are very similar (cf. Figure 3a for 300 K). The similar atomic mixing of the initially crystalline and the preamorphized sample is a consequence of the amorphization of c-Ge during ${}^{69}$Ga implantation. The amorphization threshold of Ge at 300 K is already reached at a ${}^{69}$Ga ion fluence of about 6x10${}^{13}$ cm${}^{-2}$ [13]. Considering the total fluence of 1 × 10${}^{15}$ cm${}^{-2}$ the initially crystalline sample is amorphous during more than 90% of the ion implantation time. The increasing self-atom mixing with increasing temperature observed within region I is in accord with the thermal spike model (see, e.g., Reference [1]). This model predicts that self-atom mixing mainly takes place during the relaxation stage following the collision cascade. The incident ${}^{69}$Ga ion transfers its kinetic energy through collisions to the Ge host atoms resulting in locally molten regions. With increasing temperature of the sample, the volume of these areas and the time interval, the temperature exceeds the melting point, increases. Thus, atomic mixing within these regions increases with temperature. As already mentioned above, RBS/C analysis revealed that the c-Ge (a-Ge) sample implanted at 523 K (region II) remains crystalline (amorphous). The recovery of the implantation-induced damage in c-Ge is due to dynamic annealing. This term describes a variety of processes such as the recrystallization of liquid zones and amorphous pockets and the annihilation of vacancies and self-interstitials. In the particular case of the c-Ge sample shown in Figure 3c it must be assumed that the thermal spikes formed during implantation completely recrystallize within a short relaxation period after ion impact. Within the framework of the thermal spike model, the higher mixing of the amorphous structure compared to its crystalline counterpart can be explained by the lower heat transport capacity [19] and the reduced melting point of a-Ge (965 K) compared to c-Ge (1210 K) [20,21]. The c-Ge structure shows a more pronounced intermixing for *x* > 200 nm at both 300 and 523 K than the a-Ge structure. Since ${}^{69}$Ga implantation was performed at nearly 0${}^{\circ }$ incidence and c-Ge remains crystalline at 523 K, channeling effects lead to higher penetration depths of Ga and stronger Ge mixing in c-Ge than in a-Ge for *x* > 200 nm. This is also illustrated by the comparison to the depth profile of the nuclear energy deposition. It is obvious that at *x* > 200 nm, the nuclear energy deposition is higher in c-Ge than in a-Ge. The a-Ge samples implanted at 300 and 523 K reveal a similar atomic mixing for *x* > 200 nm. This indicates similar structures of the samples, most of the time during implantation. Finally, in region III the self-atom mixing in the initially amorphous sample drops to the level of the crystalline sample. At 623 K the atomic mixing in a-Ge (c-Ge) is strongly reduced (slightly enhanced) compared to the value at 523 K. As found by RBS/C, the initially amorphous sample turns crystalline during implantation at 623 K. It is very likely that the recrystallization of a-Ge proceeds by solid phase epitaxy (SPE). This can be concluded from the experimental results on the recrystallization velocity in dependence on temperature [22]. From these data, it follows that at 623 K, the SPE regrowth process is sufficiently fast to recrystallize the entire amorphous isotope structure, whereas at 523 K, the recrystallization velocity is still too low. The difference observed in atomic mixing of a- and c-Ge at 623 K can be explained with the higher mixing efficiency of a-Ge compared to c-Ge until SPE of a-Ge is completed. Due to similar atomic masses the atomic mixing caused by the nuclear energy deposition of ${}^{69}$Ga and ${}^{70}$Ge ions should be nearly identical. This is confirmed by the fact that the results of ${}^{70}$Ge implantations continue the trend obtained by ${}^{69}$Ga implantation concerning the temperature dependence of atomic mixing (cf. Figure 4).

### 2.2. Flux and Temperature Dependence—${}^{28}$Si Implantation

#### 2.2.1. Experimental

**Figure 5:**
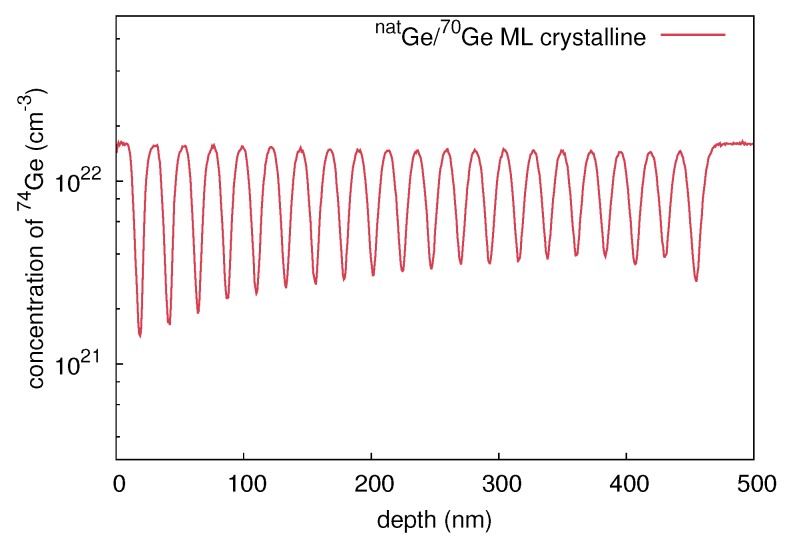
${}^{74}$Ge concentration depth profiles measured by SIMS. The crystalline multilayer structure grown by MBE contains 20 bilayers of ${}^{\mathrm{nat}}$Ge and ${}^{70}$Ge with a layer thickness of about 10 nm.

Using MBE a (mono)crystalline Ge isotope multilayer structure was grown on top of a (100)-oriented n-type Ge substrate with a specific resistivity of 40 Ωcm. The stack consists of 20 alternating bilayers (${}^{\mathrm{nat}}$Ge/${}^{70}$Ge)${}_{20}$ with an individual isotope layer thickness of about 10 nm (cf. Figure 5). In order to achieve atomic mixing 30 keV ${}^{28}$Si${}^{2+}$ ions (which corresponds to 60 keV ${}^{28}$Si${}^{+}$) were implanted using a focused ion beam (FIB) system. The direction of the ion beam was nearly perpendicular to the sample surface. The FIB spot had a diameter in the order of 100 nm. During the FIB implantation, the beam was scanned meanderlike over an area of 200 × 200 μm${}^{2}$. The scan was performed step by step with a certain pixel dwell time (PDT) and with a distance between the centers of the pixels of about 50 nm. The term pixel denotes the region irradiated by the FIB spot during one step. The FIB implantation was performed at 300 and 523 K using an ion fluence of 1 × 10${}^{15}$ cm${}^{-2}$. Changing the PDT allows a variation of the ion flux which is effectively used in the implantation (cf. Reference [18]). Two extreme cases were studied: (i) Each pixel was irradiated only once at the nominal flux of about 10${}^{18}$ cm${}^{-2}$ s${}^{-1}$ and the desired fluence was achieved by choosing the corresponding PDT. (ii) A constant PDT of 0.5 μs was used, and many repetitions of the beam scan yielded the desired fluence. In this case the effective flux was about 5 × 10${}^{11}$ cm${}^{-2}$ s${}^{-1}$. Because of the small beam spot size and the high thermal conductivity of Ge, the heating of the sample due to the FIB implantation can be neglected.

#### 2.2.2. Results and Discussion

**Figure 6:**
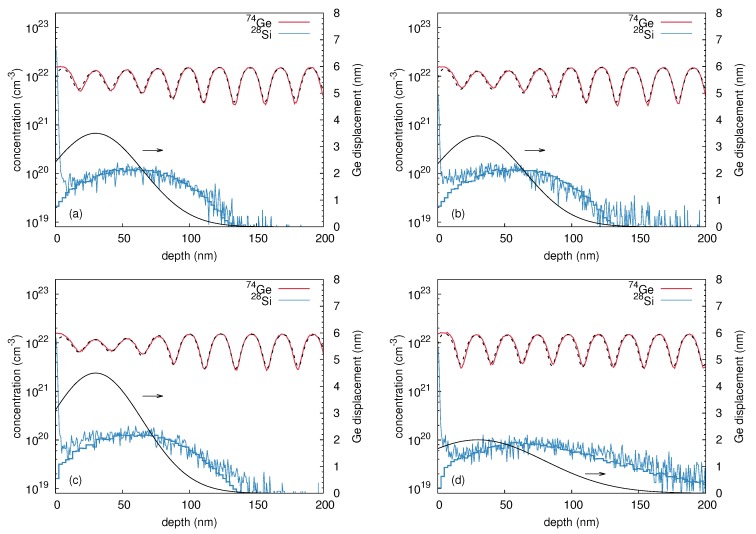
${}^{74}$Ge concentration profiles (red) measured by SIMS, after ${}^{28}$Si implantation with 60 keV, at temperatures of (**a**,**b**) 300 K and (**c**,**d**) 523 K with (**a**,**c**) a high ion flux of 10${}^{18}$ cm${}^{-2}$ s${}^{-1}$ and (**b**,**d**) a low ion flux of 5 × 10${}^{11}$ cm${}^{-2}$ s${}^{-1}$. The best fits obtained by the convolution integral analysis are shown by black dashed lines. The lower black lines show the corresponding Ge displacement function σ(x) and are referred to the right axis. The ${}^{28}$Si implantation profiles are shown in blue, together with the results of Crystal-TRIM simulations.

Figure 6 shows SIMS concentration profiles of ${}^{74}$Ge and ${}^{28}$Si after 60 keV ${}^{28}$Si implantation at 300 and 523 K, and at two different fluxes (10${}^{18}$ cm${}^{-2}$ s${}^{-1}$ and 5 × 10${}^{11}$ cm${}^{-2}$ s${}^{-1}$). Due to the relatively low ion energy only the upper part of the layer stack is affected by ion-beam-induced atomic mixing. Using Equations (2)–(4) the measured ${}^{74}$Ge depth distributions are fitted with the displacement profiles σ(x) given in the respective diagrams. The figures demonstrate that at 300 K the atomic mixing is very similar for implantations with high and low ion flux. In contrast, implantation at 523 K with a flux of 10${}^{18}$ cm${}^{-2}$ s${}^{-1}$ causes a stronger atomic mixing compared to the atomic mixing at 300 K. The low flux implantation at 523 K shows the smallest intermixing. Raman spectroscopy measurements reveal that the sample implanted at 523 K with the low ion flux of 5 × 10${}^{11}$ cm${}^{-2}$ s${}^{-1}$ remains crystalline, whereas all other samples turn non-crystalline during implantation. The detailed results of a quantitative analysis of the mixing by means of the convolution integral method based on Equations (2)–(4) are shown in Table 2. The analysis confirms that the atomic mixing for both ion fluxes is equal at room temperature, whereas at 523 K the amount of mixing strongly depends on the ion flux. At 523 K, the maximum displacement *k* obtained for $5\times 10^{11}$ cm${}^{-2}$ s${}^{-1}$ is clearly lower than for 10${}^{18}$ cm${}^{-2}$ s${}^{-1}$ and also lower compared to the results at 300 K. The concentration depth profiles of ${}^{28}$Si are all very similar, except for 523 K and a flux of $5\times 10^{11}$ cm${}^{-2}$ s${}^{-1}$. In this case the penetration depth of the Si ions is significantly increased compared to the other implantations. This is due to channeling of the implanted ions in the crystalline Ge target. The ${}^{28}$Si depth profiles are reproduced reasonably well by Crystal-TRIM simulations if in the cases of Figure 6a–c damage accumulation during implantation is assumed, whereas in the calculation of the profile in Figure 6d a crystalline target is used.

The value of the ion flux can be related to the time τ between successive ion impacts into the same region of the target. The quantity τ can be estimated by [18]

(5) $$\tau =\frac{1}{\dot{D}\sigma_{0}}$$

**Figure 7:**
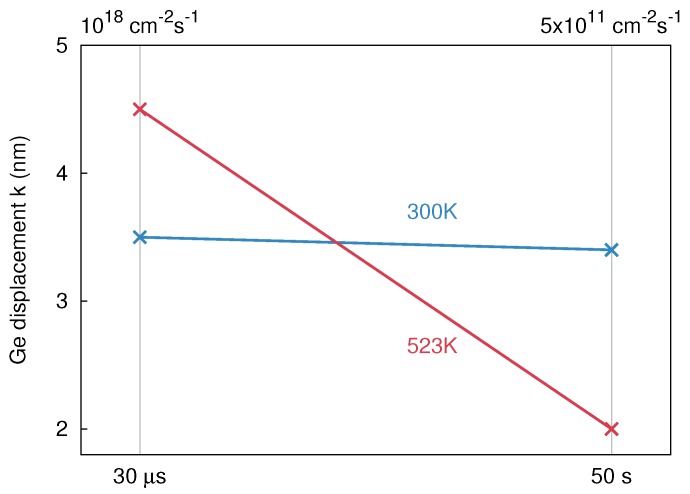
Maximum Ge displacement after 60 keV ${}^{28}$Si implantation with ion fluxes of 10${}^{18}$ cm${}^{-2}$ s${}^{-1}$ (30 μs) and $5\times 10^{11}$ cm${}^{-2}$ s${}^{-1}$ (50 s), at two different temperatures (300 and 523 K), cf. Table 2. The ion fluxes are translated into a time delay between consecutive ion impacts into the same region.

$\dot{D}$ is the ion flux and $\sigma_{0}$ is the cross section of a region with significant primary defect production given by $\sigma_{0}$ = $S_{n}$/$E_{c}$, where $S_{n}$ and $E_{c}$ are the nuclear stopping cross section of the incident ion and the critical nuclear energy deposition (per atom) for defect formation, respectively. The value of $E_{c}$ should be lower than the nominal displacement energy $E_{d}$ since most atomic displacements are formed in a region of a collision cascade where the target structure is no longer perfect. Assuming that $E_{c}$ = 0.25$E_{d}$ (cf. Reference [23]) and a displacement threshold of 15 eV the time between consecutive impacts is about 30 μs and 50 s for the high and the low ion flux, respectively (cf. Figure 7). This suggests that the damage induced by a single ion impact at 523 K is dynamically annealed within 50 s, before the next cascade affects the same region. On the other hand, a time delay of 30 μs is not sufficient to anneal the induced damage. At 300 K, the damage accumulation shows that even a time delay of 50 s between consecutive ion impacts is not long enough for dynamic annealing.

The temperature dependence of ion beam mixing illustrated in Figure 7 can be explained by the thermal spike model. At a flux of 10${}^{18}$ cm${}^{-2}$ s${}^{-1}$ the samples implanted at 300 and 523 K turn amorphous during implantation due to damage accumulation. At the higher temperature of 523 K the thermal spike lasts longer compared to 300 K. As a consequence a stronger mixing takes place at 523 K compared to 300 K. The structures irradiated at 300 and 523 K with a flux of 5 × 10${}^{11}$ cm${}^{-2}$ s${}^{-1}$ differ. As already discussed above, a-Ge has a lower thermal conductivity and a lower melting point than c-Ge. Therefore, the amorphous structure irradiated at 300 K shows a stronger mixing than the crystalline structure at 523 K. Present results are in accord with the experimental findings on the temperature dependence of atomic mixing in Ge studied by broad beam ion implantation (cf. Section 2.1.2). The ion flux used in that study is nearly the same as the low flux in the FIB implantations. The time delay between ion impacts into the same volume is there also about 50 s. It should be mentioned that plots similar to Figure 7 can be found in previous publications on the flux dependence of ion-beam-induced damage formation [18,23]. Instead of the maximum atomic displacement, in these papers a quantity characterizing the damage level is shown versus the time between consecutive ion impacts into the same region.

## 3. Ion-Beam-Induced Atomic Mixing in Silicon

### 3.1. Temperature and Dopant Dependence—${}^{69}$Ga, ${}^{70}$Ge, and ${}^{75}$As Implantation

#### 3.1.1. Experimental

**Figure 8:**
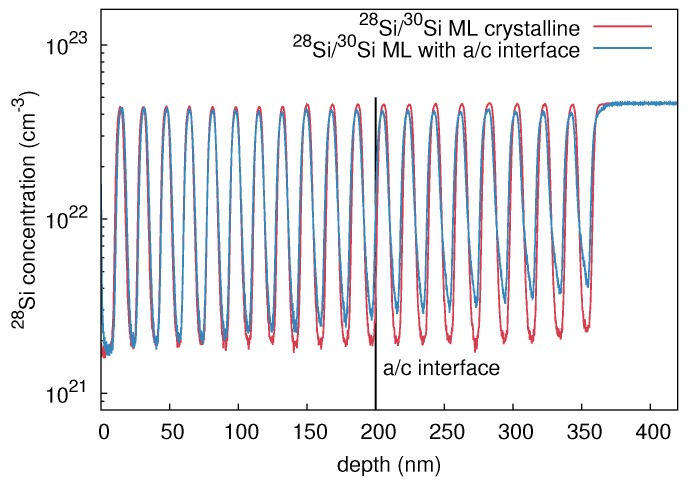
${}^{28}$Si concentration depth profiles measured with SIMS before (red) and after preamorphization (blue). The preamorphization was performed by 150 keV ${}^{28}$Si ion implantation at a fluence of 3 × 10${}^{14}$ cm${}^{-2}$. Reproduced from [Radek, M.; Bracht, H.; Liedke, B.; Böttger, R.; Posselt, M. Ion-beam induced atomic mixing in isotopically controlled silicon multilayers. *J. Appl. Phys.* **2016**, *120*, 185701.], with the permission of AIP Publishing.

(Mono)crystalline (${}^{28}$Si/${}^{30}$Si)${}_{20}$ isotope multilayers were grown by means of MBE on a (100)-oriented p-type Si wafer with a specific resistivity of about 4 cm. The thickness of each isotopically enriched layer is approximately 10 nm. Amorphous structures were prepared by implantation of ${}^{28}$Si${}^{+}$ ions into crystalline (${}^{28}$Si/${}^{30}$Si)${}_{20}$ multilayers at 77 K. Thereby, an ion energy of 150 keV, fluence of 3 × 10${}^{14}$ cm${}^{-2}$, and current of 200 nA were applied. RBS/C analysis revealed the formation of an amorphous layer of 200 nm thickness below the surface. SIMS was performed before and after preamorphization to determine the corresponding concentration depth profile of the Si isotopes. Figure 8 illustrates the ${}^{28}$Si concentration profiles of the as-grown crystalline and preamorphized isotope structures. The ${}^{28}$Si profile of the amorphous structure shows only a slightly stronger broadening than the crystalline structure for depths above 100 nm. The increasing broadening for depths above 100 nm is caused by surface roughening during SIMS profiling rather than due to the preamorphization implantation.

Samples with crystalline and preamorphized Si isotope multilayers were implanted by ${}^{70}$Ge${}^{+}$, ${}^{69}$Ga${}^{+}$, or ${}^{75}$As${}^{+}$ ions, at temperatures between 153 and 973 K, in order to investigate ion-beam-induced atomic mixing as function of temperature and dopant type. In all cases the direction of the incident ion beam was perpendicular to the (100) surface, and the implantations were performed at an energy of 180 keV, fluence of 3 × 10${}^{15}$ cm${}^{-2}$, and current of 200 nA. After implantation, the samples were rapidly quenched by cooling the sample holder with liquid nitrogen. Implantation of ${}^{70}$Ge and ${}^{69}$Ga was performed both into the crystalline and preamorphized isotope structures, whereas ${}^{75}$As was only implanted into crystalline samples.

#### 3.1.2. Results and Discussion

**Figure 9:**
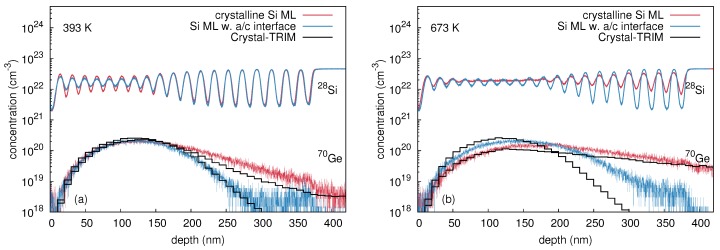
Concentration profiles of ${}^{28}$Si and ${}^{70}$Ge in initially crystalline (red) and preamorphized (blue) (${}^{\mathrm{nat}}$Si/${}^{28}$Si)${}_{20}$ multilayer structures measured by SIMS after implantation with 180 keV Ge, a fluence of 3 × 10${}^{15}$ cm${}^{-2}$, and at temperatures of (**a**) 393 K and (**b**) 673 K. The numerical prediction of the ${}^{70}$Ge implantation profiles by Crystal-TRIM calculations is shown by black histogramms.

Figure 9a,b show the ${}^{28}$Si concentration depth profiles in the initially crystalline (c-Si) and the preamorphized (a-Si) samples after ${}^{70}$Ge implantation at 393 and 673 K, respectively. Also, for direct comparison, the corresponding ${}^{70}$Ge implantation profiles are illustrated. A significant intermixing of the (${}^{28}$Si/${}^{30}$Si)${}_{20}$ multilayers is observed after ${}^{70}$Ge implantation at both temperatures compared to Figure 8. Figure 9a clearly reveals that ${}^{70}$Ge implantation at 393 K into the amorphous region (first 200 nm below the surface) causes a stronger intermixing of the host atoms than ${}^{70}$Ge implantation into the crystalline structure at the same temperature. In contrast, ${}^{70}$Ge implantation at 673 K (cf. Figure 9b) shows a stronger broadening in the crystalline than in the preamorphized structure. In order to understand the structure and temperature dependence of the ion beam mixing, additional information about structural changes induced by the implantation process is necessary. RBS/C measurements revealed a partial amorphization of the initially crystalline Si sample after implantation at 393 K and showed that the amorphous layer in the preamorphized sample increases in thickness by about 20 nm. RBS/C analysis also indicates that after implantation at 673 K the c-Si sample remains crystalline and the a-Si structure is fully recrystallized. The structural state of the samples after implantation at 393 and 673 K is also reflected by the shape of the ${}^{70}$Ge implantation profiles. At 393 K the similarity of the Ge profiles in the initially crystalline sample and in its preamorphized counterpart for depth up to 150 nm (see Figure 9a) is due to the partial amorphization found by RBS/C. Results of Crystal-TRIM simulations are consistent with the preceding discussion. In the case of 393 K damage accumulation during implantation into crystalline and preamorphized samples is assumed, and a good agreement with the SIMS data for ${}^{70}$Ge is obtained. At a temperature of 673 K the assumption of an initial amorphous layer of 200 nm thickness leads to a shallower Ge range profile than measured by SIMS since the sample is recrystallized during implantation. On the other hand, the Crystal-TRIM profile determined for the initially crystalline structure is in reasonable agreement with the experimental results.

**Figure 10:**
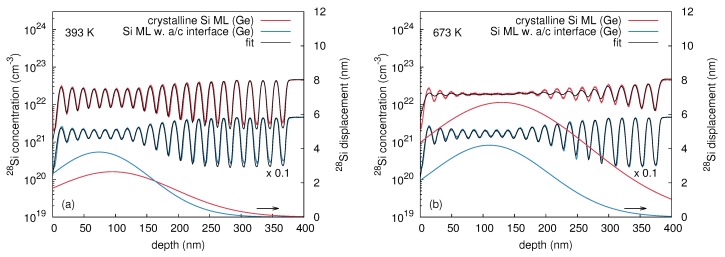
Best fits (black solid lines) based on Equations (2)–(4) to the implantation induced atomic mixing profiles of initially crystalline (red) and preamorphized (blue) Si at (**a**) 393 K and (**b**) 673 K. The ${}^{28}$Si concentration profiles in crystalline and preamorphized Si are differently scaled for clarity. The lower solid lines show the corresponding Si displacement function σ(x) and are referred to the right axis. The parameters of σ(x) for all Si samples investigated in this work are summarized in Table 3. Reproduced from [Radek, M.; Bracht, H.; Liedke, B.; Böttger, R.; Posselt, M. Ion-beam induced atomic mixing in isotopically controlled silicon multilayers. *J. Appl. Phys.* **2016**, *120*, 185701.], with the permission of AIP Publishing.

In order to describe the mixing of the matrix atoms quantitatively, the convolution integral method (cf. Section 2.1.2) was used. Figure 10a,b show best fits to the experimental ${}^{28}$Si concentration profiles obtained after ${}^{70}$Ge implantation at 393 and 673 K. The corresponding values of the displacement function σ(x) are also depicted (right ordinate axis). At 393 K the stronger intermixing of the a-Si sample compared to the c-Si structure is expressed by a higher Si displacement for depth below 150 nm. On the other hand, the displacement is similar for depths exceeding 150 nm. The displacement functions deduced from the analysis of the atomic mixing at 673 K quantifies the stronger intermixing of the c-Si structures compared to the a-Si sample. Interestingly, only the a-Si sample is accurately described by the displacement function provided by the convolution integral method. The displacement function obtained for the c-Si sample does not fully describe the depth dependent intermixing of the isotope multilayers. For example, deviations between experiment and calculated intermixing are observed for depths smaller 60 nm and at depths between 180 and 280 nm. This deviation demonstrates that the depth dependent atomic mixing within this crystalline structure cannot be modeled with a symmetric gaussian displacement function. All crystalline samples implanted at temperatures T ≥ 673 K reveal this kind of deviation. Nonetheless, the maximum displacement remains a good measure for further analysis.

**Figure 11:**
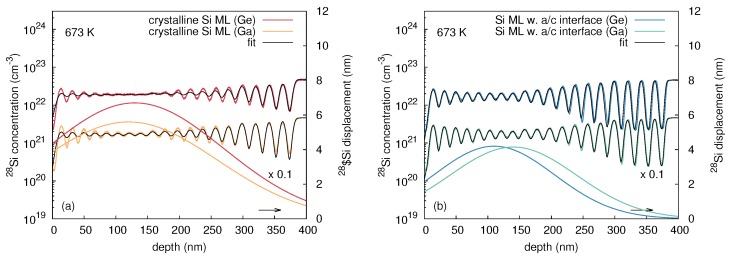
Best fits (black solid lines) to the concentration profiles of initially (**a**) crystalline and (**b**) preamorphized Si after implantation with ${}^{70}$Ge and ${}^{69}$Ga ions at a temperature of 673 K. The presentation of the data is similar to Figure 10. Reproduced from [Radek, M.; Bracht, H.; Liedke, B.; Böttger, R.; Posselt, M. Ion-beam induced atomic mixing in isotopically controlled silicon multilayers. *J. Appl. Phys.* **2016**, *120*, 185701.], with the permission of AIP Publishing.

Figure 11 demonstrates the impact of doping on atomic mixing at 673 K. Ion beam mixing caused by ${}^{69}$Ga implantation into c-Si is less pronounced compared to ${}^{70}$Ge implantation although the implantation parameters are the same and the atomic masses of both elements are very similar (see Figure 11a). On the other hand, ${}^{70}$Ge and ${}^{69}$Ga implantation in the preamorphized structure does not show a significant difference in ion-beam mixing as demonstrated in Figure 11b.

**Figure 12:**
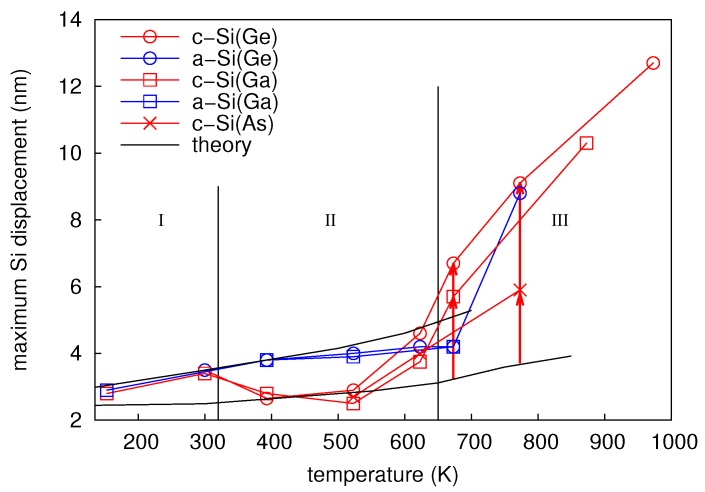
Temperature dependence of the maximum Si displacement: The mixing of the initially crystalline samples is shown by the red symbols. The results for the preamorphized samples are marked in blue. Three different ion species were used for implantation: ${}^{70}$Ge (circles), ${}^{69}$Ga (squares), and ${}^{75}$As (crosses). Three temperature regimes (I, II and III) with different mixing characteristics are identified. The black solid lines show the temperature dependence of atomic mixing in Si via thermal spikes predicted by molecular dynamics (MD) calculations. The red arrows emphasize significant differences between the black lines and experimental atomic mixing of crystalline Si for temperatures in regime III. In contrast to Ge (cf. Figure 4), ion-beam mixing in Si is described by thermal-spike mixing up to 523 K. Above this temperature, atomic mixing in the respective Si structures deviates from the thermal-spike model.

The parameters of the convolution integral method that best describe the experimental concentration profiles after ${}^{70}$Ge, ${}^{69}$Ga, and ${}^{75}$As implantation are summarized in Table 3. Figure 12 depicts the values for the maximum displacement *k* of Si atoms shown in Table 3 as function of temperature. Data corresponding to initially crystalline and preamorphized samples are marked in red and blue, respectively. Depending on the damage state revealed by RBS/C after implantation, the temperature dependence of atomic mixing can be divided into three regimes. Within the temperature regime I with T ≤ 300 K, all samples are amorphous after implantation. This is expected, because the ion fluence used in the experiments is well above the amorphization threshold of Si at these temperatures [24,25,26]. The amount of mixing increases with increasing temperature, but is independent of the initial structure and the implanted ion species. Within regime II (320 to 650 K), RBS/C shows no significant structural change within the samples after implantation, i.e., the initially crystalline samples remain crystalline and the preamorphized samples still show an amorphous layer. However, RBS/C measurements reveal a decreasing thickness of the amorphous layer in the preamorphized samples with increasing temperature indicating epitaxial recrystallization during irradiation. Similar to regime I, the amount of mixing in the preamorphized samples slightly increases with increasing temperature and is independent of the ion species. On the other hand, the amount of atomic mixing in the c-Si samples first decreases compared to region I, and finally increases for T > 523 K. No impact of the ion species on atomic mixing in the preamorphized sample is observed, whereas a clear impact of doping on the mixing efficiency in the crystalline structure is observed for T > 523 K. Implantation with either ${}^{69}$Ga or ${}^{75}$As leads to a lower atomic mixing compared to Ge. For temperatures above 650 K (regime III) RBS/C reveals that all samples are crystalline after implantation. This regime is characterized by an increasing atomic mixing with increasing temperature independently of initial structure or ion species. However, the amount of mixing is lower for implantation of the p-type dopant Ga or n-type dopant As compared to the isovalent Ge. Since all samples are crystalline after implantation, a similar amount of mixing may be expected for the same ion species. However, the mixing at 673 K significantly differs for the crystalline and initially amorphous isotope sample (cf. Figure 12). The disparity observed at 673 K demonstrates that the crystallization of the amorphous structure during implantation is not a spontaneous, but a thermally activated process. Full crystallization likely occurred just before the implantation was completed. Accordingly, an amorphous layer was present during most of the implantation time leading to the different mixing behavior compared to higher temperatures. At 773 K, the red and blue circles are nearly at the same position. Obviously, at this temperature the epitaxial recrystallization takes place very fast.

The ion-beam-induced atomic mixing in region I (see Figure 12) can be explained by the thermal spike model. With increasing sample temperature, the volume excited by the impinging ions and the time interval, the temperature exceeds the melting point, increases. As a consequence, atomic mixing within regime I increases with temperature. Regime II shows a significant difference in the amount of mixing between the initially crystalline and the preamorphized samples, i.e., the amount of mixing is higher in preamorphized compared to initially crystalline samples for temperatures below 600 K. This mixing behavior is explained by the lower melting point and reduced thermal conductivity within the amorphous structure [27] that leads to larger and longer lasting thermal spikes and thus to a stronger mixing. A similar or even higher mixing in the crystalline compared to the amorphous structure, which is observed for T ≥ 623 K cannot be explained with the thermal spike model. Furthermore, the dopant dependence, which is evident for the crystalline samples at T ≥ 500 K is not expected by this model. Therefore, additional mechanisms contribute to ion-beam-induced atomic mixing in crystalline silicon for temperatures above 500 K. It is assumed that ion-beam induced enhanced self-diffusion (IBIESD) is the relevant process. The relaxation of ion-beam induced cascades is known to form complex defect structures within the target material (cf., e.g., [28]). Accordingly, native defects formed in the course of the relaxation process can induce an additional intermixing at higher temperatures provided these defects are highly mobile and exist in sufficiently large concentrations. Such native defects are expected to cause an enhanced self-diffusion that becomes visible in the broadening of the crystalline isotope structure.

## 4. Ion-Beam-Induced Atomic Mixing in Silicon-Germanium

### 4.1. Temperature Dependence—${}^{70}$Ge Implantation

#### 4.1.1. Experimental

**Figure 13:**
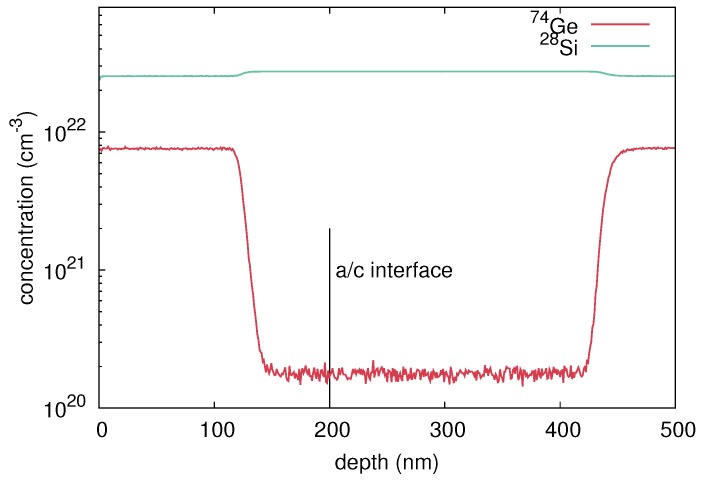
${}^{74}$Ge and ${}^{28}$Si concentration profiles measured by SIMS in a crystalline Si${}_{0.55}$Ge${}_{0.45}$ isotopically enriched sandwich structure grown by MBE. The distributions of ${}^{74}$Ge and ${}^{28}$Si atoms in the preamorphized sample are nearly identical. The vertical line marks the depth of the amorphous-crystalline interface in the preamorphized sample.

MBE was used to prepare isotopically enriched sandwich structures ${}^{\mathrm{nat}}$Si${}_{0.55}$${}^{\mathrm{nat}}$Ge${}_{0.45}$(150 nm)/ ${}^{28}$Si${}_{0.55}$${}^{70}$Ge${}_{0.45}$(300 nm)/${}^{\mathrm{nat}}$Si${}_{0.55}$${}^{\mathrm{nat}}$Ge${}_{0.45}$. Preamorphized samples were obtained by implantation of ${}^{70}$Ge${}^{+}$ ions at an energy of 200 keV, a fluence of 10${}^{14}$ cm${}^{-2}$, an ion current of 200 nA, and at a temperature of 77 K. RBS/C analysis revealed the formation of an amorphous layer of about 200 nm thickness below the surface, i.e., only the interface between the ${}^{\mathrm{nat}}$Si${}_{0.55}$${}^{\mathrm{nat}}$Ge${}_{0.45}$ top layer and the ${}^{28}$Si${}_{0.55}$${}^{70}$Ge${}_{0.45}$ layer below is affected by the amorphization. Depth profiles of ${}^{74}$Ge and ${}^{28}$Si measured by SIMS are shown in Figure 13 for the crystalline structure. The profiles in the preamorphized samples are very similar. In order to investigate ion-beam-induced mixing 350 keV ${}^{70}$Ge${}^{+}$ ion implantation into crystalline and preamorphized SiGe samples was performed at temperatures between 300 and 773 K, at a fluence of 3 × 10${}^{15}$ cm${}^{-2}$, and at nearly normal incidence. The distribution of the ${}^{74}$Ge atoms before and after implantation was measured by SIMS.

#### 4.1.2. Results and Discussion

**Figure 14:**
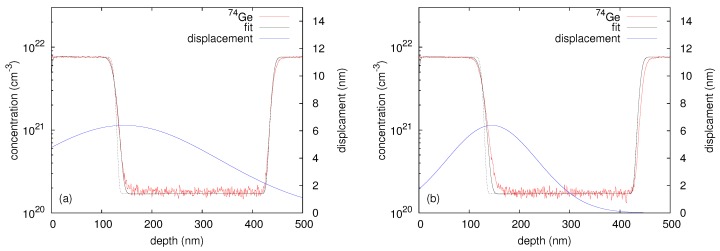
${}^{74}$Ge profiles in initially (**a**) crystalline and (**b**) preamorphized Si${}_{0.55}$Ge${}_{0.45}$ sandwich structures measured by SIMS after implantation of 350 keV ${}^{70}$Ge, at a fluence of 3 × 10${}^{15}$ cm${}^{-2}$ and at 523 K. The ${}^{74}$Ge distribution before ion beam mixing is depicted by the dotted lines. Best fits (black solid lines) based on Equations (2)–(4) to the implantation-induced atomic mixing profiles and the corresponding Ge displacement function σ(x) are also shown.

Figure 14 shows the ${}^{74}$Ge profiles in the initially crystalline and preamorphized structures after ion irradiation at 523 K. Implantation of 350 keV ${}^{70}$Ge${}^{+}$ leads to mixing at the upper interface of the sandwich structure. This can be easily seen by comparison with the initial ${}^{74}$Ge distribution depicted by dotted lines. There is a significant difference between the final profiles in both types of samples which is due to their different structures. RBS/C analysis shows that the initially crystalline sample remains crystalline and in the preamorphized sample the thickness of the amorphous sample does not change. The convolution integral method (cf. Section 2.1.2) was applied to quantify the atomic mixing in all cases considered. For 523 K the fits and the corresponding Ge displacement function σ(x) are shown in Figure 14.

**Figure 15:**
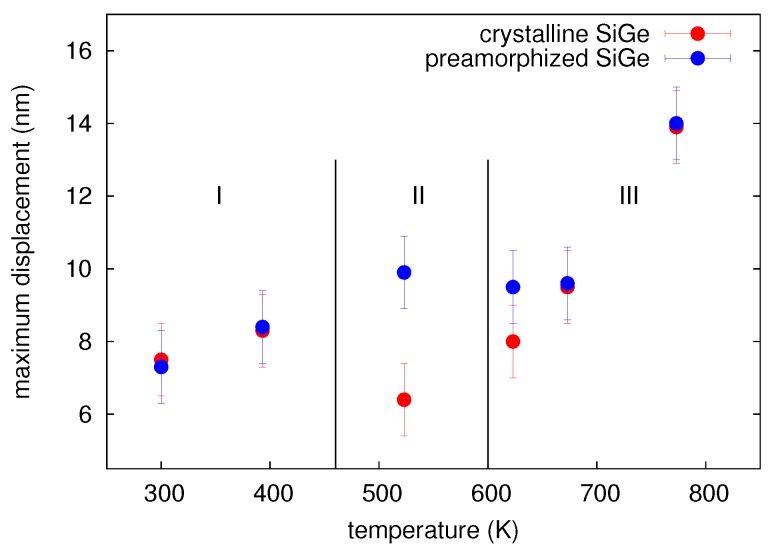
Temperature dependence of the maximum Ge displacement in initially crystalline (red) and preamorphized (blue) SiGe isotope sandwich structures. Three temperature regimes are identified based on the crystal structure (crystalline or amorphous) observed after implantation (see text for details).

Figure 15 depicts the maximum displacement of the ${}^{74}$Ge atoms after implantation versus temperature. Similar to the elemental semiconductors Si and Ge, three different temperature regimes are identified based on the structure determined by RBS/C after implantation. Atomic mixing within regime I is independent of the initial structure. This is a consequence of the amorphization of the initially crystalline samples and an increase of the thickness of the amorphous layer in the preamorphized samples. In both cases the final thickness of the amorphous layer was about 360 nm, both at 300 and 393 K. The increasing atomic mixing with increasing temperature is likely caused by thermal-spike mixing as discussed in the case of Si and Ge. As already mentioned above within regime II the initial structure of the samples does not change. Thermal-spike mixing in crystalline structures is significantly lower compared to amorphous structures which is similar to ion-beam-induced mixing in Ge and Si. RBS/C reveals that samples implanted at 623, 673 and 773 K are crystalline after ion-beam mixing (regime III). Since at 623 K epitaxial recrystallization proceeds relatively slowly, the mixing occurs mainly in amorphous material. This leads to higher atomic displacements than in the initially crystalline sample. At 673 and 773 K the initially crystalline and amorphous structures show a nearly identical amount of mixing. Similar to the case of ion-beam-induced mixing in Si, the maximum Ge displacement within the SiGe structures shows a strong increase with increasing temperature. This similarity provides evidence of an enhanced self-diffusion contribution (IBIESD) to atomic mixing in addition to thermal-spike mixing. The above discussion indicates that ion-beam-induced atomic mixing in SiGe shows the features already known from Si and Ge where the thermal spike mixing is more similar to that in Ge while IBIESD was only found in Si.

## 5. Theoretical Investigations on the Mechanisms of Ion Beam Mixing

### 5.1. Thermal-Spike Mixing Explained by Molecular Dynamics Simulations

In order to understand the origin of the temperature dependence of ion beam mixing, molecular dynamics (MD) simulations were performed. An accelerated MD code based on a General Purpose Computation on Graphics Processing Unit (GPGPU) architecture was developed to considerably speed up the calculations. The atomic interactions were described by a Stillinger-Weber(SW)-type potential combined with the repulsive Ziegler-Biersack-Littmark potential [29] at small interatomic distances. For studies of atomic mixing in Si the original parametrization of the SW potential [30] was applied, whereas for Ge the potential parameters from Posselt and Gabriel [31] were used, but with λ = 21.0. The electronic energy loss of fast moving atoms, which leads to electronic excitations in the target, was treated by applying a velocity dependent frictional force to all atoms with a kinetic energy above 10 eV, according to the Lindhard-Scharff model [32]. To characterize atomic mixing the quantity *Q* is introduced

(6) $$Q=\frac{\sum_{i}^{N}{\left({\bar{r}}_{i}^{f}-{\bar{r}}_{i}^{s}\right)}^{2}}{6C_{0}},$$

where ${\bar{r}}_{i}^{s}$ is the time-averaged position of atom *i* before the begin of a collision cascade, and ${\bar{r}}_{i}^{f}$ is the corresponding position at a given time after the start of the cascade. The time-averaging is necessary in order to separate the cascade induced displacement of atoms from the thermal vibrations at the different temperatures to be considered. In all cases an averaging over 50 ps is performed, which is well above the oscillation period of atomic vibrations. $C_{0}$ denotes the atomic density of the target material. Compared to the expression used by Nordlund et al. [10], *Q* is not normalized with respect to the deposited nuclear energy. Instead, *Q* defined in Equation (6) increases with ion energy. A direct quantitative comparison between experimental and calculated ion-beam mixing is not possible, since the number of target atoms and the kinetic energy of the primary knock-on atom (PKA) considered in thermal-spike simulation are both limited due to computational restrictions. However, in the following it is shown that the temperature dependence of thermal-spike mixing can be modeled reasonably well within the accuracy of the selected models for atomic interactions and electronic energy loss.

**Figure 16:**
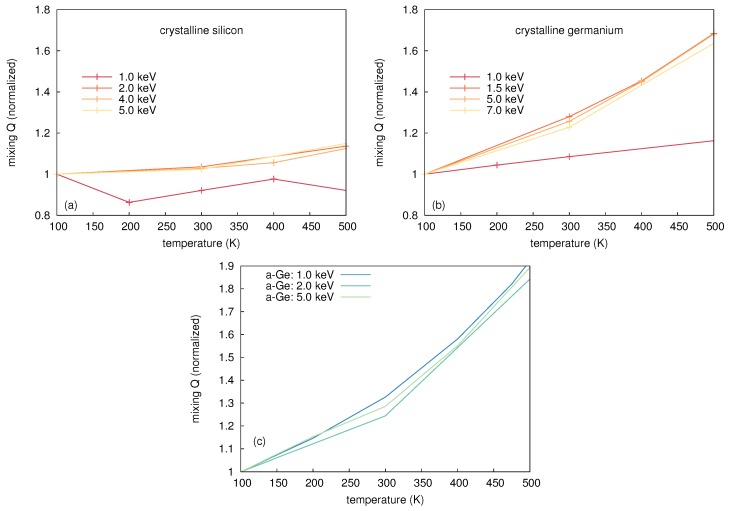
Temperature dependence of thermal-spike mixing in (**a**) crystalline Si; (**b**) crystalline Ge; and (**c**) amorphous Ge, for different energies of primary-knock-on-atoms (PKAs) deduced from molecular dynamics calculations. The atomic-mixing parameter *Q* is normalized to the mixing observed at 100 K. For PKA energies above 2 keV, the temperature dependence of *Q* is very similar in the three cases. Thermal spike mixing in amorphous Si shows a behavior very similar to that of amorphous Ge. (**a**,**b**) were reproduced from [Radek, M.; Bracht, H.; Liedke, B.; Böttger, R.; Posselt, M. Ion-beam induced atomic mixing in isotopically controlled silicon multilayers. *J. Appl. Phys.* **2016**, *120*, 185701.], with the permission of AIP Publishing.

In this work a simulation cell containing 36 × 36 × 36 unit cells of Ge or Si atoms, i.e., 373,248 atoms, was considered. Three-dimensional periodic boundary conditions were applied and the outer two unit cells were coupled to a heat bath. This ensures a realistic modeling of heat dissipation from the cascade affected region into the bulk. The use of such a thermostat has the additional benefit of damping the pressure waves induced by the sudden stopping of atoms with high velocity. Crystalline and amorphous structures were considered in order to be consistent with the experiment. The amorphous structure was prepared by quenching from the liquid phase using the method suggested by Luedtke and Landman [33]. Si or Ge atoms (PKAs) were started with kinetic energies of a few keV, at random positions and with a direction pointing to the cell center. These energies are significantly lower than the experimentally realized ion energy to meet computational restrictions of the simulation cell. High kinetic energies above some keV would mainly result in binary collisions, since at these energies the energy transfer is limited to very small impact parameters. Computer simulations of ion-beam-induced atomic mixing within the framework of the binary collision approximation, where only two atoms are involved in the interaction at any time, cannot explain any temperature dependence. When the kinetic energy decreases, the energy transfer at larger impact parameters becomes important, i.e., many-body collisions cannot be longer neglected (cf., e.g., Robinson and Torrens [34]). These collective interactions lead to the formation of thermal spikes, and MD is the suitable theoretical method to study this phenomenon. However, in MD simulations the kinetic energy of the PKA cannot be chosen arbitrarily. Figure 16 shows the temperature dependence of the total atomic mixing *Q* in crystalline Si as well as in crystalline and amorphous Ge for different PKA energies. In this figure *Q* is normalized to its value at 100 K to compare the relative change of mixing with increasing temperature. For energies above 2 keV, the predicted temperature dependence of atomic mixing is very similar for both c-Si and c-Ge. In contrast, the relative change in mixing in a-Ge does not show any PKA energy dependence for the investigated energies. Due to preceding considerations PKA energies above 2 keV were considered in the MD simulations in order to obtain meaningful results on the temperature dependence of atomic mixing in crystalline and amorphous structures.

#### 5.1.1. Thermal-Spike Mixing in Ge

**Figure 17:**
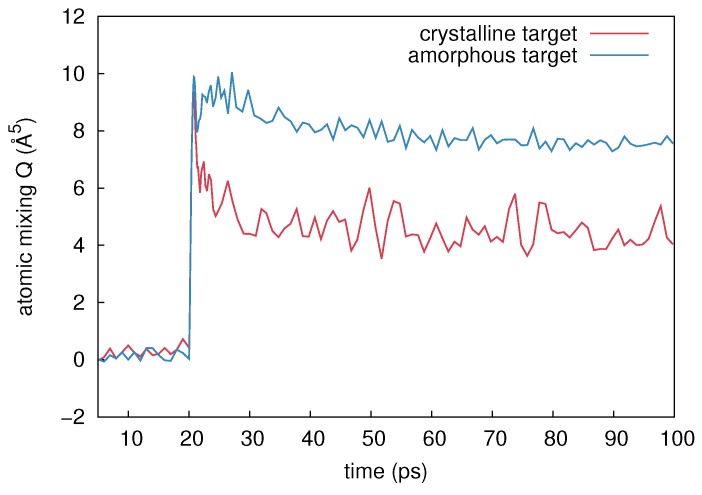
Temporal evolution of mixing after initiating a 2 keV self-atom cascade in Ge at *t* = 20 ps. The mixing in crystalline Ge is marked by red color, whereas the mixing in the amorphous structure is shown in blue.

Atomic mixing in crystalline and amorphous Ge was investigated at different ambient temperatures ranging from 100 to 823 K. In Figure 17 a typical time evolution of *Q* at 100 K caused by a 2 keV cascade is shown. After 20 ps of equilibration the cascade was initiated. A steep increase in atomic mixing is observed right after the initialization of the cascade. For *t* > 21 ps the mixing in a-Ge (blue) significantly differs from that in c-Ge (red). During the relaxation phase (21–40 ps) the mixing in the amorphous target decreases from about *Q* = 10 Å${}^{5}$ to about *Q* = 8 Å${}^{5}$. The crystalline structure shows a more pronounced decrease from *Q* = 10 Å${}^{5}$ to *Q* = 4 Å${}^{5}$. For *t* > 40 ps, no significant change in mixing is observed in both cases. These results are in full agreement with former MD calculations of Nordlund et al. [10] who found that the magnitude of mixing in a-Ge is about a factor of 2 higher than in c-Ge. Table 4 lists the numerical results of atomic mixing in c- and a-Ge at different temperatures and two PKA energies. The values were obtained by averaging over up to 30 different individual simulations for each temperature, structure, and energy. The simulations indicate that the amount of mixing in c- and a-Ge increases with increasing temperature and increasing PKA energy. Amorphous Ge always shows a stronger mixing compared to its crystalline counterpart.

To analyze the contribution of thermal spike mixing to the overall mixing measured in the simulations, the number of liquid atoms is identified during the relaxation phase of the cascade. An atom is labeled liquid during the simulation, if the average kinetic energy of itself and its nearest neighbors is above the melting temperature according to the equipartition theorem

(7) $$E_{kin}>\frac{3}{2}k_{B}T_{m}\;.$$

**Figure 18:**
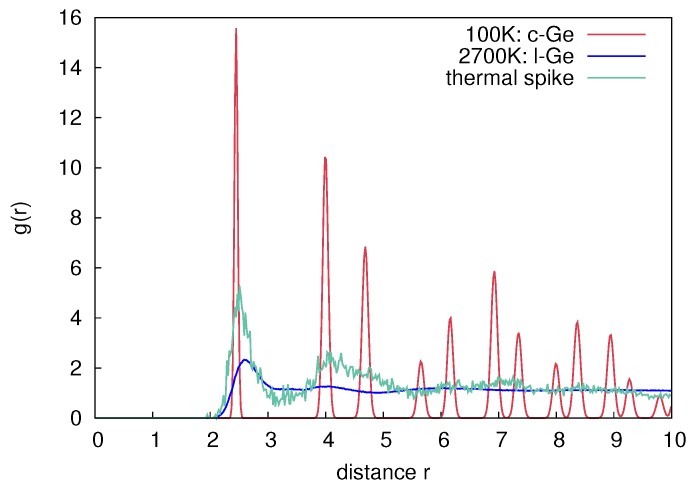
Radial distribution function of crystalline Ge at 100 K (red), liquid Ge at 2700 K (blue), and atoms within a thermal spike volume (green). The thermal spike region was determined by the amount of atoms that have a kinetic energy above the melting point according to the equipartition theorem.

Since this theorem is only valid for systems consisting of a few thousand atoms in equilibrium, which is not fulfilled in highly dynamical cascades, at least for the first few picoseconds, the values only represent a qualitative estimate. However, looking at the radial distribution function (RDF) of atoms selected with the criterion above, a liquid like state is evident. Figure 18 shows a comparison of RDFs of c-Ge (red), liquid Ge (l-Ge) at 2700 K (blue), and from atoms selected with the thermal spike criterion (green). It is evident from Figure 18, that the RDF of the thermal spike is similar to the RDF of the l-Ge, i.e., only the first two peaks are distinguishable.

Table 5 shows the maximum number of liquid atoms $n_{l}$ at different temperatures for c- and a-Ge, for a PKA energy of 5 keV. Furthermore, the integrated number of liquid atoms $n_{l}^{*}$, the atomic mixing caused by the liquid atoms $Q_{l}$, as well as the ballistic mixing $Q_{b}$ (with *Q* = $Q_{l}$ + $Q_{b}$) are listed. Ballistic mixing is calculated from the mean-square displacement of atoms with a kinetic energy above the displacement threshold energy $E_{d}$ of Ge (15 eV). As soon as the kinetic energy drops below $E_{d}$, further displacement caused by this atom no longer counts as ballistic contribution. The values given in Table 5 are averaged over up to 30 individual simulations per temperature and structure. The results show, that the number of liquid atoms increases with temperature both in c-Ge and a-Ge, as well as the mixing caused by liquid atoms, whereas the contribution of ballistic mixing remains constant within the error margins.

**Figure 19:**
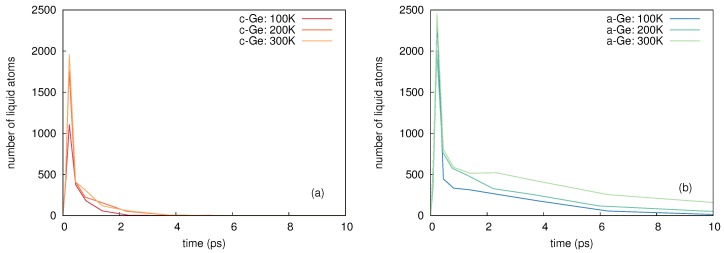
Representative temporal evolution of liquid atoms ($n_{l}$) at temperatures of 100, 200 and 300 K in (**a**) crystalline and (**b**) amorphous Ge induced by 5 keV self-atom cascades.

Figure 19 shows a representative comparison of the time evolution of the maximum number of liquid atoms $n_{l}$ in c-Ge and a-Ge during the cascade relaxation for a PKA energy of 5 keV at 100, 200 and 300 K. It is obvious, that the number of liquid atoms in a-Ge is higher compared to that in c-Ge. Furthermore the number of liquid atoms decreases significantly slower with time in a-Ge compared to c-Ge. This leads to a higher contribution of mixing in a-Ge compared to c-Ge.

The results of MD simulations confirm that with increasing temperature the amount of mixing increases. In particular the increase of the number of liquid atoms and of the associated mixing due to liquid atoms indicate, that the temperature dependence of atomic mixing observed in MD simulations is mainly mediated by thermal spike mixing. Only a small fraction of the overall mixing is caused by ballistic events, which does not show any significant temperature dependence according to the results given in Table 5. A comparison between the experimentally observed temperature dependence of atomic mixing and the temperature dependence derived from MD with a PKA energy of 5 keV in a-Ge and c-Ge is depicted in Figure 4. The mixing obtained from MD is scaled to the experimental data for c- and a-Ge at the respective lowest temperature, where additional thermally activated processes can be neglected. This allows a comparison of the temperature dependence of atomic mixing. An excellent agreement is observed. This suggests that the temperature dependence of ion-beam-induced atomic mixing in Ge is solely mediated by thermal spikes. In temperature region I (150 to 475 K) the theoretical curve agrees well with the experimental data for both c- and a-Ge, since the initially c-Ge is fully amorphized during ${}^{69}$Ga implantation (cf. Section 2.1.2). In temperature region II (475 to 550 K) the initially amorphous sample remains amorphous, the initially crystalline sample remains crystalline, and the a-Ge sample shows a higher mixing than the c-Ge samples. This can be also explained by results of MD simulations, revealing a larger amount of liquid atoms and a significantly higher mixing caused by liquid atoms in a-Ge compared to c-Ge during the relaxation stage of the cascade (see Table 5). Additionally, the temporal evolution of thermal spike mixing differs for crystalline and amorphous structures as demonstrated by Figure 17. The mixing reaches a maximum in both structures and then decreases within a few picoseconds. This decrease is related to the thermalization of pressure waves caused by the abrupt stopping of the PKA. In crystalline structures an additional mechanism affects the time evolution of atomic mixing: Due to the undisturbed lattice surrounding the thermal spike region, the periodic crystalline potential reaches into the molten region and forces the free moving atoms to fall back to a location near their original equilibrium position. This process causes a further decrease of the atomic mixing in crystalline compared to amorphous structures as demonstrated by Figure 17 for *t* > 23 ps. Finally, in temperature region III both the initially crystalline and amorphous samples are found to be crystalline (cf. Section 2.1.2) and the experimental data follow the theoretical trend obtained for the temperature dependence of atomic mixing in c-Ge. Furthermore, it is obvious that the explanation of the flux dependence of ion-beam-induced mixing in Ge given in Section 2.2.2 is supported by the above results of MD simulation on thermal spike mixing.

#### 5.1.2. Thermal-Spike Mixing In Si

Atomic mixing in crystalline and amorphous Si was studied at temperatures between 100 and 700 K using PKAs with an initial kinetic energy of 5 keV. For each temperature and structure up to 30 simulations were performed in order to obtain good statistics. The results given in Table 6 clearly show a very similar trend as obtained for thermal-spike mixing in Ge: The mixing increases with temperature and *Q* is considerably larger for a-Si than for c-Si. The MD data were scaled to experimental results at the respective lowest temperature and depicted by the black lines in Figure 12. The figure illustrates that the atomic mixing in the preamorphized and the initially crystalline structures is accurately reproduced for temperatures up to 523 K. Above this temperature MD simulations for amorphous structures overestimate the experimentally observed mixing, whereas the simulations for the crystalline structures strongly underestimate the experimental results. RBS/C revealed that at 623 K the preamorphized sample is partially crystallized. Although at 673 K the preamorphized sample is fully crystalline after implantation the recrystallization proceeds relatively slowly. As a consequence in both samples the mixing efficiency is reduced due to the increased crystalline fraction compared to the fully amorphous structure used in the simulation. The observed recrystallization process is due to ion-beam-induced enhanced crystallization that was reported for silicon at temperatures above 473 K [35]. The mixing of the initially crystalline samples exceeds the theoretical results for temperatures above 600 K. This deviation is attributed to ion-beam-induced enhanced self-diffusion (IBIESD) which is considered in detail in the next section.

### 5.2. Modeling of Ion-Beam-Induced Enhanced Self-Diffusion (IBIESD) in Si by Reaction-Diffusion Equations

The high energy deposition by incident ions causes concentrations $C_{X}$ of defects *X* that are several orders of magnitude higher than their concentrations $C_{X}^{eq}$ under thermal equilibrium. Self-diffusion in solids is controlled by the product $C_{X}D_{X}$ of defect concentration and its diffusivity $D_{X}$ (cf., e.g., [36]). This shows that only mobile defects can contribute to IBIESD. At first sight, it would be obvious to consider the contribution of single vacancies *V* and self-interstitials *I*, because these defects are mobile at room temperature [37] and also control Si self-diffusion at high temperatures [38]. Computer simulations demonstrate that only few isolated Frenkel pairs are produced by heavy-ion induced cascades in the keV regime [39,40]. On the other hand, complex defect clusters of vacancies and self-interstitials [41] are not expected to mediate self-diffusion since these clusters are fairly immobile. A highly mobile and simple defect cluster discussed in the literature is the Si di-interstitial $I_{2}$ [42,43,44]. Molecular dynamics simulations show that $I_{2}$ defects are formed during the relaxation of ion-induced cascades and during the dynamic annealing of implantation-induced damage [45,46]. Therefore, very high transport capacities ($C_{I_{2}}D_{I_{2}}$) can be achieved during implantation and concurrent damage annealing. In order to model the experimentally observed temperature dependence of ion beam mixing in crystalline Si for temperatures above 600 K, a contribution of IBIESD to the overall atomic mixing is described by a system of coupled partial differential equations

(8) $$\begin{array}{ccc} \frac{\partial C_{I_{2}}\left(x,t\right)}{\partial t}-D_{I_{2}}\frac{\partial^{2}C_{I_{2}}\left(x,t\right)}{\partial x^{2}} & = & A\times k_{0}\left(x\right)-k_{-}\left(C_{I_{2}}\left(x,t\right)-C_{I_{2}}^{eq}\right) \end{array}$$

(9) $$\begin{array}{ccc} \frac{\partial C_{\mathrm{Si}}\left(x,t\right)}{\partial t}-\frac{\partial }{\partial x}D_{\mathrm{Si}}\left(x,t\right)\frac{\partial C_{\mathrm{Si}}\left(x,t\right)}{\partial x} & = & 0\;. \end{array}$$

The Si self-diffusion coefficient under irradiation is assumed to be mainly mediated by di-interstitials, i.e., $D_{\mathrm{Si}}\left(x,t\right)\approx D_{I_{2}}C_{I_{2}}\left(x,t\right)/C_{I_{2}}^{eq}$ = $D_{I_{2}}^{\mathrm{SD}}$. The ratio $C_{I_{2}}\left(x,t\right)/C_{I_{2}}^{eq}$ represents the supersaturation of $I_{2}$ and $C_{\mathrm{Si}}$ determines the depth- and time-dependent ${}^{28}$Si concentration of the (${}^{28}$Si/${}^{30}$Si)${}_{20}$ multilayer. $k_{0}$ denotes the depth-dependent production rate of $I_{2}$ whereas $k_{-}$ describes its dissociation rate

(10) $$k_{-}=\nu \exp\left(-\frac{E_{b}}{k_{B}T}\right)$$

with the Debye frequency $\nu (\approx 10^{13}\;s^{-1})$ and the binding energy $E_{b}$ of $I_{2}$. In the simulation of IBIESD, only the parameter *A* that represents the amplitude of the normalized production rate $k_{0}\left(x\right)$ is considered as a real fitting parameter. The normalized $k_{0}\left(x\right)$ profile itself is assumed to be equal to the normalized depth profile of nuclear energy deposition obtained from Crystal-TRIM calculations, for 180 keV ${}^{70}$Ge implantation into (100)-oriented Si with an incidence angle of 0${}^{\circ }$ with respect to the surface normal. The contribution of thermal-spike mixing to the overall mixing is taken into account by an appropriate initial ${}^{28}$Si profile. This profile is obtained from the as-grown crystalline ${}^{28}$Si profile (cf. Figure 8) considering a broadening according to thermal spike mixing with the maximum displacement calculated by MD (cf. black line for c-Si in Figure 12), with the same depth dependence as the profile of nuclear energy deposition. Thermal spike mixing and IBIESD are assumed to not influence each other, since they occur on different time scales, i.e., thermal spike mixing happens during the relaxation phase of the cascade that takes several picoseconds, whereas diffusion phenomena happen on the time scale of minutes to hours. The surfaces of the samples are considered to maintain the equilibrium concentration of $I_{2}$, i.e., $C_{I_{2}}\left(x=0,t\right)$ = $C_{I_{2}}^{eq}$, the concentration relative to the Si-atom density $C_{0}$ is assumed to be close to zero, i.e., $C_{I_{2}}^{eq}$/$C_{0}$ = 10${}^{-13}$. Before implantation (t = 0) the system is assumed to be in thermal equilibrium, i.e., C${}_{I_{2}}$(x, 0) = C${}_{I_{2}}^{\mathrm{eq}}$ and *A* = 0. According to the MD calculations reported in the literature, the self-diffusion coefficient via $I_{2}$ is calculated for a particular temperature by [43]

(11) $$D_{I_{2}}^{\mathrm{SD}}=4.1\times 10^{-4}\exp\left(-\frac{0.38\;\mathrm{eV}}{k_{B}T}\right){\mathrm{cm}}^{2}\;s^{-1}\;.$$

Theoretical calculations suggest a binding energy, $E_{b}$ for $I_{2}$ of 1.3 eV [47] to 2.2 eV [48], depending on the assumed charge state of the di-interstitial and the method used for calculation. The simulation of IBIESD via Equations (8)–(11) is very sensitive to the choice of $E_{b}$. Best fits are obtained assuming a binding energy of 1.1 eV for all simulations. This value is close to that suggested by Lopez and Fiorentini [47], who assume a neutral defect.

**Figure 20:**
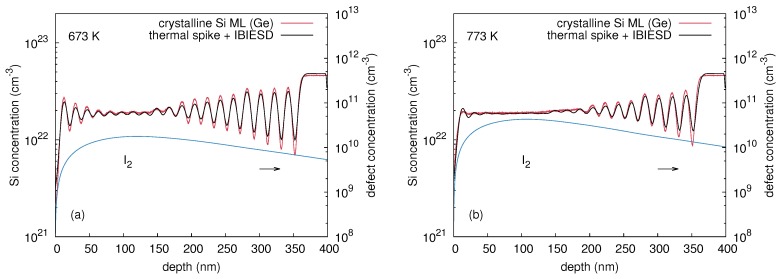
${}^{28}$Si concentration depth profile after implantation of ${}^{70}$Ge ion with an energy of 180 keV and a fluence of 3 × 10${}^{15}$ cm${}^{-2}$ (red) at (**a**) 673 K and (**b**) 773 K, in comparison to the numerical model consisting of a combination of thermal spike mixing and ion beam induced enhanced self-diffusion (IBIESD) mediated by Si di-interstitials (black). The concentration of the silicon di-interstitials is shown as blue solid line and is referred to the right axes. (**a**) was reproduced from [Radek, M.; Bracht, H.; Liedke, B.; Böttger, R.; Posselt, M. Ion-beam induced atomic mixing in isotopically controlled silicon multilayers. *J. Appl. Phys.* **2016**, *120*, 185701.], with the permission of AIP Publishing.

**Figure 21:**
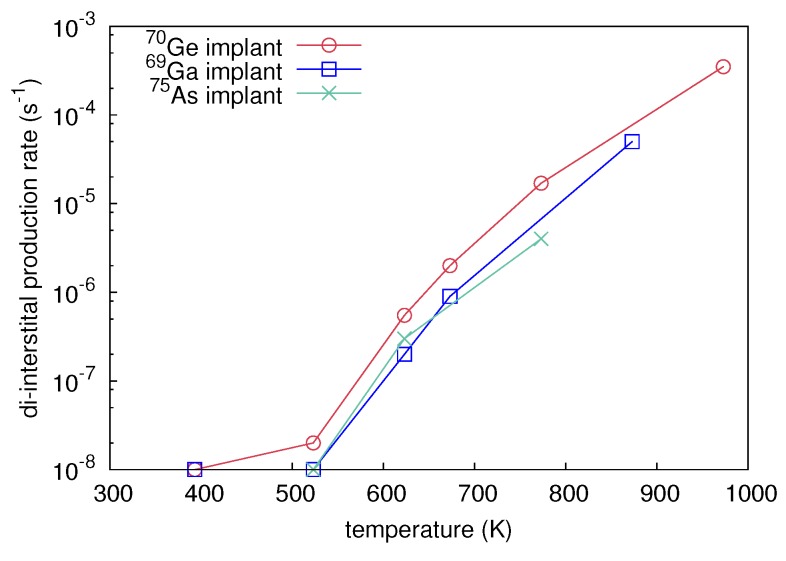
Production rate of Si di-interstitials as obtained from best fits of the ion-beam induced enhanced self-diffusion (IBIESD) model to the experimental data versus implantation temperature. Three different ion species were considered: ${}^{70}$Ge (red circles), ${}^{69}$Ga (blue squares), and ${}^{75}$As (green crosses). Reproduced from [Radek, M.; Bracht, H.; Liedke, B.; Böttger, R.; Posselt, M. Ion-beam induced atomic mixing in isotopically controlled silicon multilayers. *J. Appl. Phys.* **2016**, *120*, 185701.], with the permission of AIP Publishing.

Figure 20 demonstrates the best fit to the atomic mixing of the crystalline isotope multilayer induced by ${}^{70}$Ge implantation at 673 and 773 K based on the di-interstitial model as described above. All ${}^{28}$Si profiles obtained after ion implantation into the crystalline Si isotope multilayer are reproduced reasonably well with *A* as a fitting parameter. Figure 21 depicts the quantity *A*, i.e., the maximum production rate of di-interstitials induced by ${}^{70}$Ge, ${}^{69}$Ga, and ${}^{75}$As implantation, as function of temperature. The numerical simulations of IBIESD suggest an increasing di-interstitial production rate with increasing temperature. This causes an increasing supersaturation of di-interstitials that enhances self-diffusion. This temperature dependence of IBIESD is consistently explained by the dissolution of defect clusters that inject additional di-interstitials beside those created directly in the collision event. Present interpretation is also supported by the observed doping dependence of IBIESD. Modeling of the experimental results provides systematically lower di-interstitial production rates for both ${}^{69}$Ga and ${}^{75}$As implantation compared to ${}^{70}$Ge implantation. Obviously, dynamic annealing of damage in doped Si occurs more readily than in undoped Si leading to a lower amount of defect clusters that inject di-interstitials. This is also consistent with studies on ion-beam-induced enhanced crystallization of Priolo et al. [49,50] which show that the presence of n- or p-type dopants significantly enhances crystallization under irradiation.

## 6. Comparison of Ion-Beam-Induced Atomic Mixing in Si and Ge

**Figure 22:**
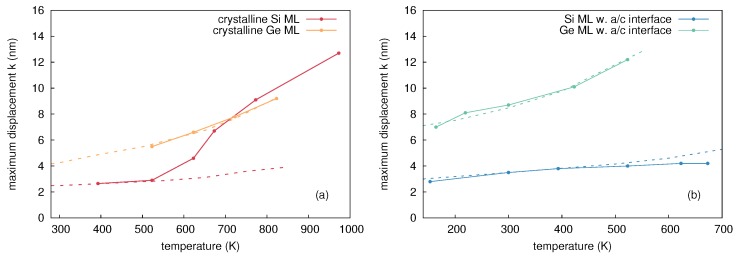
Comparison between the experimentally determined maximum atomic displacements in initially (**a**) crystalline and (**b**) preamorphized isotope multilayer structures of Si (Ge) after ${}^{70}$Ge (${}^{69}$Ga and ${}^{70}$Ge) implantation at 180 keV (310 keV) at an ion fluence of 3 × 10${}^{15}$ cm${}^{-2}$ (1 × 10${}^{15}$ cm${}^{-2}$). The contributions of thermal spike mixing calculated by molecular dynamics to the overall mixing are shown as dashed lines in the same color code as the experimental data.

**Figure 23:**
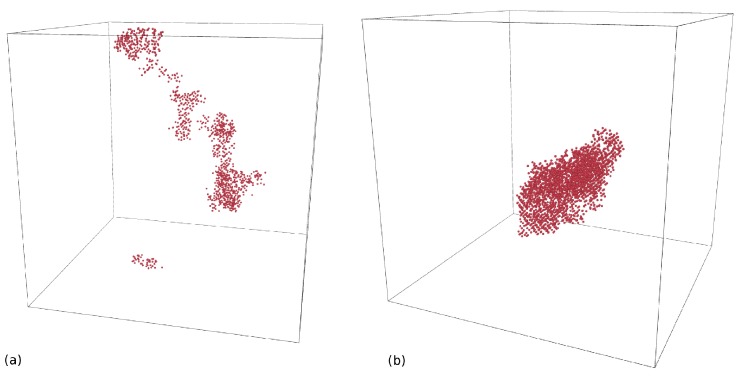
Representative simulation snapshots of thermal spikes in (**a**) Si and (**b**) Ge induced by a 5 keV self-atom cascade. The temperature during the simulation was kept at 100 K.

The different temperature dependence of ion-beam-induced atomic mixing in Si and Ge is striking. No evidence of IBIESD in Ge is observed at temperatures between 164 and 823 K, i.e., the temperature dependence of ion-beam mixing is fully described by thermal-spike mixing (cf. Figure 4). Figure 22 compares the temperature dependence of atomic mixing in Si and Ge in detail. The maximum displacement of initially crystalline and preamorphized samples are shown. The experimental data points for Si were obtained after Ge implantation where doping has no impact on atomic mixing. The results of MD simulations for thermal spike mixing are shown as dashed lines. Direct comparison of ion beam mixing in Si and Ge reveals, that not only the magnitude of mixing, but also the temperature dependence of the maximum displacement differs. In particular for *T* < 523 K, where thermal spike mixing prevails the mixing in initially crystalline and preamorphized Si, the temperature dependence is less pronounced than in Ge. Figure 23 shows representative snapshots of a thermal spike induced by a 5 keV PKA in c-Si and c-Ge. Only atoms with a kinetic energy $E_{kin}$ > 0.168 eV in the case of Ge and $E_{kin}$ > 0.2185 eV in the case of Si are shown. These energies correspond to the average kinetic energy of atoms at the melting point according to the equipartition theorem. The geometric shape of the two thermal spike volumes strongly differs. A subdivision of the whole thermal spike into smaller spikes is clearly visible in the case of Si, whereas only one large and compact thermal spike is observed in Ge. Since the scattering cross-section of a PKA in Ge is larger compared to the case of Si, the energy deposition per volume is higher and the mean free path of single energetic particle is lower. Individual thermal spikes induced by sub-cascades overlap leading to a large single thermal spike that covers the complete cascade volume. The lower surface area of the thermal spike volume together with the lower thermal conductivity of Ge compared to Si leads to slower energy dissipation away from the cascade affected region, which increases the time the thermal spike affected regions exceeds the melting point. As a consequence, the temperature dependence of ion-beam-induced atomic mixing is more pronounced in Ge compared to Si. Table 7 summarizes the maximum number of liquid atoms $n_{l}$, the integrated number of liquid atoms $n_{l}^{*}$, the mixing $Q_{l}$ caused by liquid atoms, as well as the ballistic mixing $Q_{b}$ as calculated from up to 30 individual cascade simulations per temperature and crystal structure in Si and Ge. As expected, the fraction of ballistic mixing $Q_{b}$ to the overall mixing *Q* in Si is significantly higher compared to Ge. This is due to the smaller collision cross section and the corresponding larger mean free path of single energetic particles in silicon.

Above 523 K the deviation of the maximum displacement in c-Si (cf. Figure 22a) from results of MD simulations is attributed to IBIESD by di-interstitials (cf. Section 5.2). In c-Ge the temperature dependence of ion-beam-induced atomic mixing is solely described by thermal spike mixing, i.e., no additional mechanism of mixing is evident. Obviously, mobile defects that may lead to enhanced self-diffusion are not formed at sufficiently high concentration during the implantation process. This observation is consistent with studies of damage annealing in Ge after implantation, where cascade induced defects or end-of-range defects are hardly observed [51,52]. Compared to Si the damage annealing in Ge is more efficient. This goes along with a lower injection rate of mobile native defects in Ge compared to Si, leading to an insignificant contribution of self-diffusion to atomic mixing. An enhanced diffusion of self- and boron atoms is reported for Ge under proton irradiation [53,54,55,56,57]. In contrast to heavy ion implantation considered in this work, proton irradiation mainly forms isolated Frenkel pairs without collision cascades and thermal spikes. The concentration of these isolated native point defects is then sufficiently high to enhance such a diffusion process.

## 7. Summary

Ion-beam-induced atomic mixing in initially crystalline and preamorphized isotopically modulated Ge and Si multilayers as well as in isotopically enriched SiGe sandwich samples was investigated at temperatures between 153 and 973 K, using ${}^{70}$Ge, ${}^{69}$Ga, and ${}^{75}$As ions. It is observed that the amount of mixing increases with temperature and depends on whether the target material is predominantly crystalline or amorphous during the irradiation. Additionally to the dependence on temperature the influence of ion flux and ion species was studied. Using a focused ion beam system it was shown that at elevated temperature (523 K) implantation into Ge at a very high flux (10${}^{18}$ cm${}^{-2}$ s${}^{-1}$) leads to stronger mixing than at a flux comparable to that used in standard broad beam ion implantation, while there is no flux dependence at room temperature. Implantation with various ions (Ga${}^{+}$, Ge${}^{+}$ and As${}^{+}$) revealed that at temperatures above about 623 K atomic mixing in crystalline Si depends on ion species. In Ge such dependence was not observed after implantation with Ga${}^{+}$ or Ge${}^{+}$.

Molecular dynamics simulations demonstrated that the temperature and the flux dependence of atomic mixing in Ge are solely mediated by the thermal spike mechanism. Thermal spikes are formed due to localized energy deposition of primary-knock-on atoms generated in the collision cascade of the implanted ion. If the energy density is sufficiently high, locally molten zones are formed, where atomic motion occurs faster compared to the solid state. With increasing temperature, the volume of the thermal spikes and the time, the temperature of the molten zones exceeds the melting point, increases. Thus, thermal spike mixing increases with increasing temperature. In Si thermal spike simulations are able to describe the temperature dependence of mixing up to 523 K. The number of liquid atoms and their contribution to the thermal spike mixing as extracted from molecular dynamics simulations are significantly higher in amorphous than in crystalline structures. The reduced thermal conductivity and the reduced melting point of the amorphous structures compared to fully crystalline samples lead to larger and longer lasting thermal spikes and thus to stronger atomic mixing. A direct comparison of the amount of mixing between Si and Ge reveals that Ge shows a stronger thermal spike mixing in the crystalline as well as in the amorphous state. Furthermore, a more pronounced dependence on temperature is evident in the case of Ge. The material specific scattering cross-section as well as the difference in thermal conductivity, and the efficiency of damage build-up and dynamic annealing mechanisms are identified as reasons for the disparities.

Above 523 K a systematic deviation of the temperature dependence of experimental mixing data in Si from results obtained by thermal spike simulations is found. The difference is proposed to be caused by an additional mixing mechanism called ion-beam-induced enhanced self-diffusion, which is mediated by di-interstitials created directly by the impinging ion or during the annealing of ion-beam-induced damage. Above 523 K atomic mixing in crystalline Si depends on the ion species used for implantation. The atomic mixing is reduced for both dopants Ga and As compared to the isovalent dopant Ge. This suggests a stronger annealing efficiency of defect clusters under p- and n-type doping which obviously reduces the injection rate of mobile defects that are responsible for the enhanced self-diffusion. The diffusion model considered only accounts for a contribution of di-interstitials. Despite this limitation, a reasonable good agreement between experiment and simulation is obtained. Certainly, the process of ion-beam-induced enhanced self-diffusion is more complex and involves other mobile defects and reactions with clusters that can act both as sinks and sources. In this regard, the proposed model represents the simplest approach to consistently interpret the experimental data with defect properties reported for di-interstitials.

Ion-beam-induced atomic mixing in SiGe can be qualitatively explained by the findings obtained from detailed investigations on this effect in Ge and Si. At low temperatures the thermal spike mechanism should dominate, whereas at higher temperatures ion-beam-induced enhanced self-diffusion seems to be the prevailing process of atomic mixing.

Finally, it should be mentioned that the methods applied in this work to investigate atomic mixing during ion irradiation of Si, Ge, and SiGe might be also applied to study the mechanisms of surface evolution and pattering in Si/Ge systems (cf., e.g., References [58,59,60]).

## Figures and Tables

**Table 1:** Fit parameters *k* (amplitude), *l* (position), and *m* (width) of the depth dependent σ(x) function deduced from the analysis of self-atom mixing in crystalline and preamorphized Ge isotope multilayers. The mixing of the isotope structure was induced by 310 keV ${}^{69}$Ga or ${}^{70}$Ge implantation at a fluence of $1\times 10^{15}$ cm${}^{-2}$ and temperatures ranging from 164 to 823 K.

	Sample	Ion		T (K)	*k* (nm)	*l* (nm)	*m* (nm)
regime I							
	c-Ge	${}^{69}$Ga${}^{+}$		164	7.0 ± 0.3	80 ± 5	95 ± 5
	c-Ge	${}^{69}$Ga${}^{+}$		219	8.1 ± 0.3	110 ± 5	120 ± 5
	c-Ge	${}^{69}$Ga${}^{+}$		300	8.7 ± 0.3	75 ± 5	110 ± 5
	a-Ge	${}^{69}$Ga${}^{+}$		300	8.7 ± 0.3	85 ± 5	85 ± 5
	c-Ge	${}^{69}$Ga${}^{+}$		423	9.7 ± 0.3	80 ± 5	115 ± 5
	a-Ge	${}^{69}$Ga${}^{+}$		423	10.1 ± 0.3	85 ± 5	105 ± 5
regime II							
	c-Ge	${}^{69}$Ga${}^{+}$		523	5.5 ± 0.3	110 ± 5	135 ± 5
	a-Ge	${}^{69}$Ga${}^{+}$		523	12.2 ± 0.3	85 ± 5	85 ± 5
regime III							
	c-Ge	${}^{69}$Ga${}^{+}$		623	6.6 ± 0.3	120 ± 5	110 ± 5
	a-Ge	${}^{69}$Ga${}^{+}$		623	7.5 ± 0.3	90 ± 5	118 ± 5
	c-Ge		${}^{70}$Ge${}^{+}$	723	7.9 ± 0.3	110 ± 5	150 ± 5
	c-Ge		${}^{70}$Ge${}^{+}$	823	9.2 ± 0.3	110 ± 5	150 ± 5

**Table 2:** Fit parameters *k* (amplitude), *l* (position), and *m* (width) of the depth dependent σ(x) function deduced from the analysis of atomic mixing in crystalline Ge isotope multilayers. The mixing of the isotope structure was induced by 60 keV ${}^{28}$Si implantation at two strongly different ion fluxes ($5\times 10^{11}$ cm${}^{-2}$ s${}^{-1}$, $10^{18}$ cm${}^{-2}$ s${}^{-1}$) at temperatures of 300 K and 523 K.

Ion Flux (cm${}^{-2}$s${}^{-1}$)	T (K)	*k* (nm)	*l* (nm)	*m* (nm)
$5\times 10^{11}$	300	3.4 ± 0.3	30 ± 5	35 ± 5
$10^{18}$	300	3.5 ± 0.3	30 ± 5	35 ± 5
$5\times 10^{11}$	523	2.0 ± 0.3	30 ± 5	50 ± 5
$10^{18}$	523	4.5 ± 0.3	30 ± 5	35 ± 5

**Table 3:** Fit parameters *k* (amplitude), *l* (position), and *m* (width) of the depth dependent displacement function σ(x) deduced from the analysis of self-atom mixing in crystalline (c-Si) and preamorphized (a-Si) Si isotope multilayers after ${}^{69}$Ga, ${}^{70}$Ge, and ${}^{75}$As implantation at temperatures ranging from 153 K to 973 K.

	Sample	Ion			T (K)	*k* (nm)	*l* (nm)	*m* (nm)
regime I								
	c-Si	${}^{69}$Ga${}^{+}$			153	2.8 ± 0.3	100 ± 5	100 ± 5
	a-Si	${}^{69}$Ga${}^{+}$			153	2.9 ± 0.3	85 ± 5	90 ± 5
	c-Si		${}^{70}$Ge${}^{+}$		300	3.5 ± 0.3	75 ± 5	85 ± 5
	a-Si		${}^{70}$Ge${}^{+}$		300	3.5 ± 0.3	70 ± 5	85 ± 5
	c-Si	${}^{69}$Ga${}^{+}$			300	3.4 ± 0.3	100 ± 5	100 ± 5
	a-Si	${}^{69}$Ga${}^{+}$			300	3.5 ± 0.3	70 ± 5	83 ± 5
regime II								
	c-Si	${}^{69}$Ga${}^{+}$			393	2.8 ± 0.3	130 ± 5	130 ± 5
	a-Si	${}^{69}$Ga${}^{+}$			393	3.8 ± 0.3	85 ± 5	90 ± 5
	c-Si		${}^{70}$Ge${}^{+}$		393	2.7 ± 0.3	95 ± 5	100 ± 5
	a-Si		${}^{70}$Ge${}^{+}$		393	3.8 ± 0.3	75 ± 5	85 ± 5
	a-Si	${}^{69}$Ga${}^{+}$			523	3.9 ± 0.3	100 ± 5	85 ± 5
	c-Si	${}^{69}$Ga${}^{+}$			523	2.5 ± 0.3	130 ± 5	120 ± 5
	c-Si		${}^{70}$Ge${}^{+}$		523	2.9 ± 0.3	130 ± 5	130 ± 5
	a-Si		${}^{70}$Ge${}^{+}$		523	4.0 ± 0.3	110 ± 5	75 ± 5
	c-Si			${}^{75}$As${}^{+}$	523	2.7 ± 0.3	100 ± 5	100 ± 5
	c-Si	${}^{69}$Ga${}^{+}$			623	3.7 ± 0.3	130 ± 5	95 ± 5
	c-Si		${}^{70}$Ge${}^{+}$		623	4.6 ± 0.3	130 ± 5	130 ± 5
	a-Si		${}^{70}$Ge${}^{+}$		623	4.2 ± 0.3	110 ± 5	85 ± 5
	c-Si			${}^{75}$As${}^{+}$	623	4.0 ± 0.3	95 ± 5	100 ± 5
regime III								
	c-Si	${}^{69}$Ga${}^{+}$			673	5.7 ± 0.3	85 ± 5	95 ± 5
	a-Si	${}^{69}$Ga${}^{+}$			673	4.2 ± 0.3	100 ± 5	95 ± 5
	c-Si		${}^{70}$Ge${}^{+}$		673	6.7 ± 0.3	130 ± 5	130 ± 5
	a-Si		${}^{70}$Ge${}^{+}$		673	4.2 ± 0.3	110 ± 5	95 ± 5
	c-Si		${}^{70}$Ge${}^{+}$		773	9.1 ± 0.3	130 ± 5	130 ± 5
	a-Si		${}^{70}$Ge${}^{+}$		773	8.8 ± 0.3	120 ± 5	120 ± 5
	c-Si			${}^{75}$As${}^{+}$	773	5.9 ± 0.3	95 ± 5	85 ± 5
	c-Si	${}^{69}$Ga${}^{+}$			873	10.3 ± 0.3	100 ± 5	100 ± 5
	c-Si		${}^{70}$Ge${}^{+}$		973	12.7 ± 0.3	130 ± 5	130 ± 5

**Table 4:** Thermal spike mixing *Q* (Å${}^{5}$) after initiating self-atom cascades in crystalline (c-Ge) and amorphous (a-Ge) germanium at temperatures ranging from 100 K to 823 K and PKA energies of 2 keV and 5 keV. The data are averaged over up to 30 individual simulations per energy, temperature, and structure.

T (K)	2 keV	5 keV
100: c-Ge	17.97 ± 3.23	59.41 ± 14.03
100: a-Ge	29.85 ± 3.23	91.24 ± 16.32
200: c-Ge		63.41 ± 22.93
200: a-Ge		105.18 ± 20.24
300: c-Ge	19.70 ± 2.87	74.11 ± 18.15
300: a-Ge	37.14 ± 2.87	117.33 ±15.17
400: c-Ge		85.49 ± 20.76
400: a-Ge		141.53 ± 14.26
500: c-Ge	22.50 ± 3.94	95.37 ± 20.12
500: a-Ge	50.02 ± 3.94	172.72 ± 11.35
550: c-Ge		102.13 ± 22.58
550: a-Ge		198.59 ± 31.08
650: c-Ge		117.32 ± 29.67
650: a-Ge		
723: c-Ge		142.44 ± 34.52
823: c-Ge		172.06 ± 38.29

**Table 5:** Maximum number of liquid atoms $n_{l}$, integrated number of liquid atoms $n_{l}^{*}$, the mixing caused by liquid atoms $Q_{l}$, as well as the ballistic mixing $Q_{b}$ are shown at temperatures of 100 K, 200 K, and 300 K averaged over up to 30 cascade simulations induced by 5 keV self-atoms.

T (K)	$n_{l}$	$n_{l}^{*}$	$Q_{l}\times 10^{-3}$ (Å${}^{5}$)	$Q_{b}\times 10^{-3}$ (Å${}^{5}$)
100: c-Ge	1617 ± 237	2664 ± 263	44.2 ± 9.1	13.2 ± 8.3
100: a-Ge	1972 ± 310	4055 ± 370	74.2 ± 15.3	15.2 ± 4.1
200: c-Ge	1762 ± 485	2950 ± 796	49.0 ± 17.8	13.6 ± 9.1
200: a-Ge	2194 ± 615	4953 ± 967	91.4 ± 19.4	12.5 ± 5.3
300: c-Ge	1915 ± 530	3294 ± 893	63.2 ± 19.2	9.2 ± 5.9
300: a-Ge	2106 ± 620	6073 ± 1724	96.0 ± 22.1	19.8 ± 8.9

**Table 6:** Atomic mixing *Q* for crystalline and amorphous silicon induced by 5 keV self-atom cascades at temperatures between 100 K and 700 K calculated with Equation (6).

T (K)	Target	Q $\times \;10^{-3}$ (Å${}^{5}$)
100	crystalline	53.55 ± 9.45
100	amorphous	80.56 ± 12.61
300	crystalline	54.82 ± 8.42
300	amorphous	97.95 ± 16.11
500	amorphous	116.28 ± 11.58
550	crystalline	63.22 ± 10.93
600	amorphous	127.95 ± 18.19
650	crystalline	68.60 ± 6.51
700	amorphous	152.18 ± 23.50
750	crystalline	79.03 ± 15.54

**Table 7:** Maximum number of liquid atoms $n_{l}$, integrated number of liquid atoms $n_{l}^{*}$, the mixing caused by liquid atoms $Q_{l}$, as well as the ballistic mixing $Q_{b}$ are shown at temperatures between 100 K and 300 K averaged over up to 30 cascade simulations induced by 5 keV self-atoms in silicon and germanium.

T (K)	$n_{l}$	$n_{l}^{*}$	$Q_{l}\times 10^{-3}$ (Å${}^{5}$)	$Q_{b}\times 10^{-3}$ (Å${}^{5}$)
100: c-Si	974 ± 279	1873 ± 241	10.8 ± 5.1	42.2 ± 14.4
100: a-Si	760 ± 135	3512 ± 380	32.2 ± 10.3	48.2 ± 15.7
300: c-Si	1232 ± 488	2306 ± 546	17.4 ± 6.6	37.4 ± 16.2
300: a-Si	869 ± 141	4617 ± 537	44.9 ± 14.7	52.4 ± 17.1
100: c-Ge	1617 ± 237	2664 ± 263	44.2 ± 9.1	13.2 ± 8.3
100: a-Ge	1972 ± 310	4055 ± 370	74.2 ± 15.3	15.2 ± 4.1
200: c-Ge	1762 ± 485	2950 ± 796	49.0 ± 17.8	13.6 ± 9.1
200: a-Ge	2194 ± 615	4953 ± 967	91.4 ± 19.4	12.5 ± 5.3
300: c-Ge	1915 ± 530	3294 ± 893	63.2 ± 19.2	9.2 ± 5.9
300: a-Ge	2106 ± 620	6073 ± 1724	96.0 ± 22.1	19.8 ± 8.9

## Abbreviations

The following abbreviations are used in this manuscript:

a-Ge	preamorphized (Section 2) or amorphous germanium (Section 5)
a-Si	preamorphized (Section 3) or amorphous silicon (Section 5)
FIB	focused ion beam
GPGPU	general purpose computation on graphics processing unit
IBIESD	ion-beam induced enhanced self-diffusion
MBE	molecular beam epitaxy
MD	molecular dynamics
PDT	pixel dwell time
PKA	primary knock-on atom
RBS/C	Rutherford backscattering spectrometry in channeling geometry
RDF	radial distribution function
SIMS	secondary ion mass spectrometry
SPE	solid phase epitaxy
SW	Stillinger-Weber
XTEM	cross-sectional transmission electron microscope
